# Sustainable synergistic approach to chemolithotrophs—supported bioremediation of wastewater and flue gas

**DOI:** 10.1038/s41598-024-67053-2

**Published:** 2024-07-17

**Authors:** Rachael J. Barla, Suresh Gupta, Smita Raghuvanshi

**Affiliations:** Faculty Division-1, Department of Chemical Engineering, Birla Institute of Technology and Science (BITS PILANI), Pilani, 333031 Rajasthan India

**Keywords:** Chemolithotrophs, Bubble column bioreactor, Metabolite extraction, Mass transfer, Techno-economic assessment, Thermodynamics, Biotechnology, Engineering

## Abstract

Flue gas emissions are the waste gases produced during the combustion of fuel in industrial processes, which are released into the atmosphere. These identical processes also produce a significant amount of wastewater that is released into the environment. The current investigation aims to assess the viability of simultaneously mitigating flue gas emissions and remediating wastewater in a bubble column bioreactor utilizing bacterial consortia. A comparative study was done on different growth media prepared using wastewater. The highest biomass yield of 3.66 g L^−1^ was achieved with the highest removal efficiencies of 89.80, 77.30, and 80.77% for CO_2_, SO_2_, and NO, respectively. The study investigated pH, salinity, dissolved oxygen, and biochemical and chemical oxygen demand to assess their influence on the process. The nutrient balance validated the ability of bacteria to utilize compounds in flue gas and wastewater for biomass production. The Fourier Transform–Infrared Spectrometry (FT–IR) and Gas Chromatography–Mass Spectrometry (GC–MS) analyses detected commercial-use long-chain hydrocarbons, fatty alcohols, carboxylic acids, and esters in the biomass samples. The nuclear magnetic resonance (NMR) metabolomics detected the potential mechanism pathways followed by the bacteria for mitigation. The techno-economic assessment determined a feasible total capital investment of 245.74$ to operate the reactor for 288 h. The bioreactor’s practicability was determined by mass transfer and thermodynamics assessment. Therefore, this study introduces a novel approach that utilizes bacteria and a bioreactor to mitigate flue gas and remediate wastewater.

## Introduction

Urbanization and industrialization are the two primary sources of economic, social, and political challenges affecting the quality of life throughout the world. 99% of the world’s population, according to the World Health Organization (WHO), resides in areas where WHO pollution quality requirements are not met. In 2019, it was predicted that air pollution caused 4.2 million premature deaths worldwide. Similarly, 2 million tonnes of sewage, agricultural, and industrial waste are dumped into the water sources daily (UN WWAP, 2003), equivalent to the weight of the total human population^1^. Industrialization contributes significantly to environmental degradation, and it is multiplied because of massive rural–urban migration intensifying urbanization. These migrations result from the perception of superior economic chances in industrialized, economically thriving urban areas. The rapid industrialization in and around metropolitan centers has imposed severe air and water pollution pressures on the atmosphere, as well as water bodies such as rivers, lakes, and ponds^2,3^.

Earth’s average temperature has risen by 0.6 °C up to 2019, and a further increase of 6.4 °C is forecasted between now and 2100. An increase in greenhouse gases (GHGs) such as carbon dioxide (CO_2_) and nitrogen oxides (NOx), and indirect GHGs such as sulfur oxides (SOx) are the primary reasons for global warming^4^. Emissions of CO_2_ account for 76.7% of all GHGs, while NOx and SO_2_ add another 7.9% and 2.8%, respectively. These gaseous emissions are commonly referred to as flue gas emissions, which increased by 70% between 1970 and 2024^5^. Power plants, cement factories, coal-fired plants, etc., are the primary sources of flue gas emissions. The acidic gases such as carbon monoxide (CO), nitric oxide (NO), and sulfur dioxide (SO_2_) emitted from industries result in the formation of smog and acid rain. These pollutants are linked to various skin and eye infections after human contact^6^.

Various methods have been developed to reduce the emission of exhaust gases in industrial settings. Absorption and adsorption are the most used post-combustion methods for reducing the harmful effects of flue gas emissions from industries. These methods can offer a higher removal efficiency, adsorbent recycling, and solvent regeneration. However, the increased concentrations of SO_2_ and NOx necessitate substantial amounts of energy and solvent, corrode equipment, and reduce efficiency^7,8^. Similarly, the membrane technique can be used in various gas separation applications and has promising results. However, energy requirements and equipment expenses are extensive, and SO_2_ inhibition has a negative impact on the membrane process^9,10^. Nonetheless, a new bio-mitigation approach uses chemolithotrophs to reduce flue gas emissions from industries. Chemolithotrophic bacteria play a role in transforming CO_2_ into carbonate (CO_3_^2−^), which is consumed by the bacteria as the inorganic carbon source. This process aids in reducing CO_2_, thereby preventing its harmful effect on the environment and thus supporting endeavors to alleviate climate change^11^. Certain bacteria can oxidize sulfur and nitrogen compounds, such as SO_2_ and NO, and utilize them as an inorganic energy source^12^. It is feasible to transform the toxic compounds into less detrimental forms by using the metabolic processes of these bacteria. This procedure can be implemented in specific industrial environments to diminish the release of flue gas, a significant factor in air pollution and acid rain formation.

The industries mentioned above also release significant quantities of liquid effluents or wastewater. About 80% of industry wastewater is estimated to be discharged worldwide without proper treatment. The WHO estimates that by 2050, roughly half of humanity will reside in areas with inadequate water supplies^1^. Consequently, wastewater remediation aims to restore the water to its natural cycle after removing solids, liquids, and microbes that could cause disease^13^. The primary and secondary treatment procedures in typical wastewater treatment remove the bulk of the larger particles and organic debris, respectively. The tertiary treatment eliminates leftover contaminants from the treated water^14^. These treatments entail a combination of chemical, physical, and biological procedures.

The biological process methodology is advantageous to the other chemical and physical methods as it is economical, energy-intensive, and environmentally friendly. Chemolithotrophs can take up organic and inorganic nutrients from flue gas and wastewater sources to supplement their growth substrate for development. Increases in removal efficiency, biomass, and lipid productivity can all be attributed to a mixotrophic cultivation system^15^. The chemical oxygen demand: nitrogen: phosphorus (COD:N:P) ratio of around 100:5:1 and 250:5:1 is ideal for wastewater treatment for aerobic and anaerobic systems, respectively^16^. Compared to the nitrogen content, wastewater's carbon content is relatively low. Hence, the required carbon source could be acquired from the CO_2_ from the flue gas for bacterial growth^12^. Agricultural, industrial, and municipal liquid waste effluents can be investigated as low-cost growing media for chemolithotrophs. Adding these sources and the flue gas can be an advantage for the proliferation of chemolithotrophs by making an abundance of nutrients available^17^. Besides being ecologically benign, cost-effective, and sustainable, the treatment process should consider the origin and composition of flue gas and wastewater^4^.

The use of omic technologies to study bacterial lipid metabolism under different stress conditions has recently gained importance. Metabolomics investigations on bacteria subjected to environmental stresses have documented variations in the abundance of particular genes, proteins, or metabolites^18^. Metabolomics studies aim to analyze comprehensive groups of molecules that are crucial for supporting important regulatory elements (such as genes, proteins, metabolites, and their interactions) and mechanisms that reflect the physiological aspects of bacterial growth and adaptation to changes in cultivation^19^. Wastewater and flue gas in a medium is treated biologically by chemolithotrophs through intricate biochemical reactions. Under the harmful effects of pollutants, a bacterial consortium consisting of multiple species can evolve a detoxification mechanism. The resistance mechanisms that their cells can develop are the following: the creation of extracellular barriers, extracellular sequestration and active movement of pollutants, and intracellular sequestration and reduction or oxidation of compounds^20^. Lipids, proteins, and carbohydrates are synthesized at the expense of cellular metabolism during mitigation. These lipids and carbohydrates obtained from the bacterial biomass have further applications, including developing commercial products and generating bioenergy^21^. Hence, the metabolomics of the synthesized metabolites are performed to identify the mechanisms followed by the bacteria for growth under stress conditions of flue gas and wastewater.

This study examines the bioremediation efficiency of the chemolithotrophs for the treatment of industrial flue gas and wastewater. A simulated flue gas and wastewater from a sewage treatment plant have been coherently treated in a 20 L pilot-scale bubble column bioreactor utilizing mixotrophic chemolithotrophs. The chemolithotrophs were grown in flue gas and wastewater and were analyzed for growth kinetics, nutrient removal efficiency, and biomass composition. The effect of pH, salinity, dissolved oxygen (DO), biological oxygen demand (BOD), and COD was studied for the bioreactor system. The total nutrient assimilation approximation was made to establish a relationship between the dual process (flue gas mitigation and wastewater remediation) and the growth of the chemolithotrophs. The proteinogenic and lipid productivity analysis of the biomass achieved was done using the Fourier Transform–Infrared Spectrometry (FT–IR), Gas Chromatography–Mass Spectrometry (GC–MS), and nuclear magnetic resonance (NMR) approaches. The mass transfer and thermodynamics assessments established favorable conditions for metabolic reaction and product formation. A techno-economic analysis of the process proved the efficiency of the bioreactor. Thus, a sustainable and efficient process was created to utilize waste discharges and nutrient sources to cultivate biomass simultaneously.

## Materials and methods

### Experimental design and feed characterization

**Figure 1:**
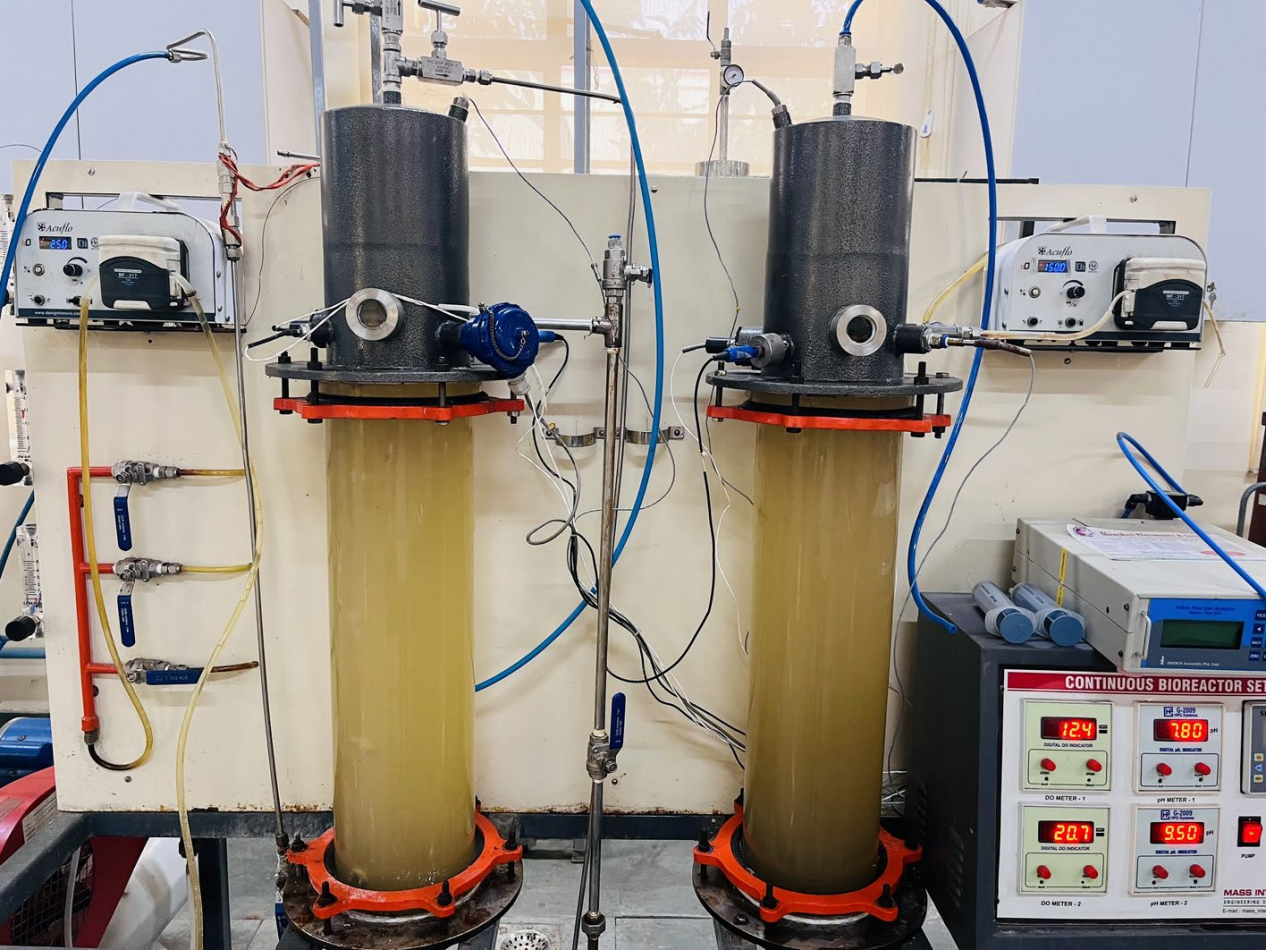
Bioreactor setup for flue gas mitigation and wastewater remediation.

A pilot-scale bioreactor was used to conduct the semi-continuous experiments. The flue gas was fed in continuous mode, whereas the wastewater was in batch mode in the reactor. The bioreactor comprised two glass columns, each holding 10 L of aqueous medium plus 2 L of head space. The reactor was run with both columns connected in series with a working volume of 20 L and a total volume of 24 L, including headspace. Figure 1 depicts the actual representation of the bubble column bioreactor. Before beginning the experiment, the column was sealed entirely and rendered gas- and liquid-tight. Each column stood at a height of 24 inches and a width of 6.5 inches. The column base was fitted with a ring sparger and a perforated sieve plate for sparging tiny, homogeneous bubbles and avoiding biomass buildup on the sparger. A minimal salt media (MSM) tank was located at the system's rear. The medium was pumped up from the base of the column and into the reactor using a peristaltic pump. The gaseous mixture was constantly delivered at a volumetric flow rate of 2 L min^−1^ from the mixing chamber to the column’s intake. An attached rotameter passed the gas mixture into the column. There was a constant flow of media between the two columns, and the gas collected in the first column's headspace was continuously supplied to the second column from the bottom. A second column was fitted with a peristaltic pump to withdraw the liquid sample for analysis.

The simulated flue gas, ordered from Ankur Speciality Gases & Technologies Pvt Ltd., India, contained 10.4% CO_2_, 780 ppm NO, and 141 ppm SO_2_, equivalent to the effluent flue gas concentration of a typical cement industry. The cylinder obtained contained standardized and sterilized flue gas. This simulated gas was combined with air comprising 20% O_2_ in the mixing chamber. The temperature in the reactor setup was maintained at ambient conditions of 30 ± 2 °C throughout the cultivation period. The flue gas mixture was fed into the reactor with a pressure of 1 bar at a volumetric flow rate of 2 L min^−1^ and ambient temperature. The wastewater (WW) was acquired from the secondary clarifier’s discharge section of the sewage treatment facility of Birla Institute of Technology and Science (BITS), Pilani, Rajasthan, India (28.3588° N, 75.5880° E). The WW was initially sterilized at 121 °C for 30 min to kill any microorganisms present. The sterilized WW was subjected to testing on an agar medium to eliminate the possibility of any microorganisms being present in it, ensuring that only the bacteria cultivated in the inoculum were responsible for the bioremediation process. The WW was treated to primary settling to remove big, insoluble particles. The WW was next filtered with Whatman 0.22 µm cellulose membrane filter paper to eliminate any suspended particles. The standard procedures of the American Public Health Association (APHA) outlined in the experimental and analytical analysis section of the paper were used to characterize the collected WW’s initial nutrient concentration^22^. The components measured were as follows (in g L^−1^): carbonate (CO_3_^2-^), bicarbonate (HCO_3_^−^), phosphate (PO_4_^3−^), sulfite (SO_3_^2−^), sulfide (S^2−^), sulfate (SO_4_^2−^), nitrate (NO_3_^−^), nitrite (NO_2_^−^), ammonium (NH_4_^+^), BOD, COD, total dissolved solids (TDS) and trace metal elements such as nickel (Ni), zinc (Zn), and iron (Fe (II)). The initial WW nutrient concentrations are depicted in Table 1.

**Table 1:** Wastewater characteristics obtained from the STP.

Parameters	Concentration (g L^−1^)	Parameters	Concentration (g L^−1^)
pH	8.2	Nitrite	0.004
Salinity	1.38 × 10^–9^	Ammonium	0.008
Carbonate	2.5	Biological oxygen demand	0.006
Bicarbonate	0	Chemical oxygen demand	0.465
Phosphate	2.73	Nickel	0.003
Sulphite	0.25	Zinc	0.002
Sulphide	0.26	Iron	0.021
Sulphate	0.22	Total dissolved solids	0.010
Nitrate	0.27		

### Bacterial strain and culture cultivation

The bacteria employed in the experiment originated from sludge and water samples obtained from the hypersaline environment of Sambhar Salt Lake (SSL) in Rajasthan, India (26.9042° N, 75.1717° E). The site was selected for bacterial isolation due to its abundance of sulfur-oxidizing, denitrifying, and carbon-utilizing bacteria, which is essential to a successful bio-mitigation and remediation strategy^23^. All solutions were prepared with WW and autoclaved to ensure their sterile nature. Bacteria from the sludge and water samples were enriched using the selective enrichment culture method. First, the culture was grown and acclimatized in a flue gas mixture for 6 days. The grown culture was further enriched in a nutrient broth medium containing sodium chloride (5 g L^−1^), peptone (5 g L^−1^), and beef extract (1.5 g L^−1^). The enrichment was performed in an orbital shaker (OSI, Metrex Scientific, India) for 18 h at 37 °C and 120 rpm. The MSM solution in the reactor was inoculated with 2 L of the enriched culture (1.93 at OD_600nm_) to the 20  L reactor volume. The MSM for bacterial cultivation in the reactor was prepared containing the following salts (g L^−1^): Potassium Nitrate (KNO_3_)-1, Dipotassium Hydrogen Phosphate (K_2_HPO_4_)-1, Ammonium Chloride (NH_4_Cl)-0.16, Sodium Thiosulphate (Na_2_S_2_O_3_)-24.5 and Sodium Chloride (NaCl)-35^24^. The experiments were conducted in two sets: MSM with WW and only WW. The individual experiments were carried out on the same reactor. Each experiment was performed for 12 consecutive days (288 h).

The experimental approach included daily plating of the active bacterial species in the reactor, with the final day sample (12th day) being isolated and processed for species identification. The specimens were sent to Eurofins Genomics Pvt. Ltd., located in Bangalore, India, to conduct 16 s metagenomics sequencing. The metagenomic DNA was obtained from the samples using the Nucleospin Soil kit (MN) for extraction. The extracted metagenomic DNA samples were assessed using NanoDrop, which measured the A260/280 ratio and quantified with a Qubit fluorometer. The initial amplification polymerase chain reaction (PCR) was conducted using the metagenomic DNA obtained and a primer set designed for the bacterial 16S V3-V4 region. The PCR setup utilized the following primers: 16S rRNA Forward—GCCTACGGGNGGCWGCAG and 16S rRNA Reverse—ACTACHVGGGTATCTAATCC. The PCR product was electrophoresed on a 1.2% Agarose gel at 120 V for around 60 min. The library preparation process utilized primers specific to the 16 s region. The library validation was performed utilizing the Agilent Tape station kit, which incorporates the High Sensitivity D1000 Screen tape. The sequencing was conducted using the latest sequencing chemistry on the Illumina platform, resulting in 1,00,000 reads per sample.

### Experimental and analytical analysis

The gaseous and liquid sampling and analysis were performed every 24 h. A flue gas analyzer (FGA) (FGA 53X, Indus Scientific, India) was utilized to measure the flue gas concentration directly at the bioreactor's entrance and outflow. The input port of the FGA was directly connected to the column's gaseous outlet port. The FGA measures the CO_2_, and, NOx and SOx concentrations following the principle of nondispersive infrared (NDIR) and electrochemical sensors, respectively. The analyzer is supplied with standard flue gas Peltier cooler conditioning system and probe assembly. The liquid samples were withdrawn from the outlet present in the second column into sterilized sampling tubes^12^. The sample's optical density (OD) was measured using a UV–VIS Spectrophotometer (Evolution 201, Thermo Scientific, USA) at 600 nm, with the prepared MSM media acting as the standard^12^. The sample was plated on an agar medium for the colony-forming unit (CFU), which was counted with a ten-fold serial dilution^12^. All the methodologies are based on previous work performed on a laboratory-scale fermenter^12^. A pH meter (Eco Testr, Eutech Instruments, Singapore) and a Microprocessor water and soil analysis kit (MWS, Khera Instruments, India) were used to determine the sample's pH, DO, and salinity, respectively^24^. The daily sample was filtered using Whatman 0.22 µm cellulose membrane filter paper and then oven-dried at 60 °C. The filter paper's weight was measured before and after drying to estimate the biomass’s dry weight^24^. The filtrate obtained from the above was utilized to measure the dissolved CO_2_ concentration using a dissolved CO_2_ analyzer (Portable CO_2_ analyzer, OxyGuard, Denmark)^24^.

Following the standard procedure described in the literature of the American Public Health Association (APHA), the filtrate obtained from the sample was used to calculate the CO_3_^2−^, HCO_3_^-^, PO_4_^3−^, SO_3_^2−^, S^2−^, SO_4_^2−^, NO_3_^−^, NO_2_^−^ and NH_4_^+^ concentrations^22^. The closed reflux colorimetric method was used to measure the COD. The sample was mixed with a digestion solution (potassium dichromate, sulphuric acid, and mercuric sulfate) and sulphuric acid reagent (silver sulfate and sulphuric acid) and digested in a COD block digester (HI 839800, Hanna Instruments, India) at 150 °C. The absorbance of the digested sample was measured along with the reagent blank at 600 nm. The 5-day BOD estimation was done by incubating the sample in a BOD incubator (VSLIC-E110, Vertex, India) at 20 °C for 5 days^22^.

### FT–IR, GC–MS, and NMR metabolite analysis

The GC–MS (QP-2010 Plus, Shimadzu, Japan) and FT-IR (Frontier, Perkin Elmer, India) analyses were used to evaluate lipid formation during the bio-mitigation of flue gases using the biomass obtained at the end of the 288-h experimental run. The 50 mL bacterial culture was subjected to centrifugation at 10,000 rpm with a relative centrifugal force (RCF) of 9838 g-force for 20 min at 4 °C in order to prepare it for separate FT–IR, GC–MS, and NMR analyses. The biomass and supernatant obtained from centrifugation were lyophilized using a lyophilizer (ScanVac freeze-dryer, LaboGene, Scandinavia) for the FT-IR examination. After centrifugation, the obtained cell pellet was utilized as an intracellular (IC) and cell-free supernatant for extracellular (EC) product identification by GC–MS analysis. The detailed procedure followed for the FT-IR analysis and extraction of product metabolites is described in the literature^25^. The data was compared to a database of mass spectra stored in the GC–MS library (NIST-05 and Wiley-8).

The centrifuged bacterial biomass obtained from the centrifugation mentioned above was washed with a phosphate buffer (20 mM, pH 7.2) to eliminate the waste medium and prevent metabolome contamination. After three cycles of centrifugation, the final cell pellet was treated with cell lysis buffer and lysed according to the methodology described in the GC–MS analysis. After cell lysis, the solution was centrifuged for 10 min at 10,000 rpm (9838 RCF) and 4 °C, and the supernatant containing extracted metabolites was collected. The supernatant samples were lyophilized overnight in the lyophilizer. The lyophilized samples were dissolved in 0.5 mL of deuterium oxide (D_2_O) containing a chemical shift indicator (4,4-dimethyl-4-silapentane-1-sulfonic acid (DSS), 0.5 mM, pH 7.5). The Bruker AV NEO (400 MHz, Bruker, USA) NMR spectrometer was used to capture all proton NMR spectra. The acquired 1D spectra were Fourier converted at a line broadening value of 0.5 Hz, phased, and baseline adjusted in Mnova V.14 (Mestrelab research) software. Each spectrum’s ^1^H NMR chemical shifts were calibrated relative to the 0.00 ppm DSS peak. Chemical shifts in the spectrum were identified and assigned using the Mnova profiler’s 400 MHz chemical shift database and then verified with other databases to ensure accuracy^26,27^.

### Theoretical evaluations

The bacterial growth was determined by measuring the OD and dry-weight biomass. Biomass productivity (*P*_max_, g L^−1^ h^−1^) and specific growth rate (*µ*, hour^-1^) were calculated using equations mentioned in the previous study^25^. The performance of the bubble column reactor for the flue gas bio-mitigation and WW remediation was evaluated by calculating the removal efficiency (*RE*, %), elimination capacity (*EC*, g L^−1^ h^−1^), inlet loading (*IL*, g L^−1^ h^−1^), and residence time (*RT*, minutes). The RE, EC, IL, and RT were evaluated using equations mentioned in the previous study^24^.

Further, the bio-fixation rate ($R_{gas}$, g L^−1^ h^−1^) of individual gas in the flue gas mixture was calculated by Eq. (1) using the previously obtained values^12^.

1 $$R_{gas} = C_{g} \times P_{max} \times \frac{{M_{gas} }}{{M_{g} }}$$

where $M_{gas}$ is the molecular weight of the gas_,_ and *M*_g_ is the atomic weight of carbon, nitrogen, or sulfur. *C*_g_ is the biomass’s carbon, nitrogen, or sulfur content and was determined using a Vario MACRO Cube elemental analyzer (Elementar Analysen Systeme GmbH, Germany). The WW remediation efficiency was calculated by the biomass yield, $Y_{X/S}$ (g cells/g substrate), and nutrient utilization efficiency (g L^−1^ h^−1^) depicted in Eqs. (2 and 3)^12^:

2 $$Y_{X/S} = - \frac{\Delta X}{{\Delta S}}$$

3 $$Rate \;of \;nutrient \;utilization = \frac{{N_{out} - N_{in} }}{\Delta t}$$

$\Delta X$ and $\Delta S$ are the differences in cell and substrate concentration (g L^−1^), respectively. *N*_in_ and *N*_out_ are the initial and final nutrient concentrations (g L^−1^), and ∆*t* is the time interval, respectively.

### Mass transfer characteristics

The gas–liquid mass transfer enables the transfer of the substrate solute from the gas to the liquid phase. Out of the 4 gases involved in the experiment, NO, O_2,_ and CO_2_ are sparingly soluble in water (NO: 0.032 g L^−1^, O_2_: 0.041 g L^−1^, and CO_2_: 1.496 g L^−1^ at 1 atm and 25 °C). Therefore, the liquid phase mass transfer resistance dominates over the gas phase resistance^28^. Hence, the dynamic method was utilized to calculate the volumetric mass transfer coefficient of NO, O_2,_ and CO_2_ in the system using Eqs. (4 and 5)^29^.

4 $${\text{For}}\;{\text{ gas}}\;{\text{ out}}\;{\text{ period}}:\;C_{Lo} - C_{L} = \left( {q_{o} C_{x} } \right) t$$

5 $${\text{For }}\;{\text{gas - in }}\;{\text{period}}:C_{L} = - \frac{1}{{k_{L} a}} \frac{{dC_{L} }}{dt} + \left( {C_{L}^{*} - \frac{{q_{o} C_{x} }}{{k_{L} a}}} \right)$$

where *C*_Lo_ is the initial gas concentration, *C*_L_ is the dissolved gas concentration, *q*_o_ is the specific gas uptake rate by the bacteria, *C*_x_ is the biomass concentration, *t* is time, *k*_L_a is the liquid phase volumetric mass transfer coefficient, and *C*_L_^*^ is the saturation condition of the gas in the aqueous media under temperature and pressure conditions.

In comparison to NO and CO_2_, SO_2_ is highly soluble in water (94 g L^−1^ at 1 atm and 25 °C), and hence the gas phase resistance dominates. Hence, the gas phase analysis method was used to calculate the volumetric mass transfer coefficient of SO_2_ in the system using Eq. (6)^30^.

6 $$k_{G} a = \frac{{Q_{G} }}{{V_{L} }} ln\left( {\frac{{SO_{2} ,in}}{{SO_{2} ,out}}} \right)$$

$k_{G} a$ is the gas phase volumetric mass transfer coefficient, *Q*_G_ gas flow rate (L min^−1^), *V*_L_ is the reactor volume (L), $SO_{2} ,in$ and $SO_{2} ,out$ are the inlet and outlet SO_2_ concentrations (ppm), respectively.

### Statistical analysis

The analyses were conducted in triplicate. The results are presented as the mean value ± the standard error of the mean for the assays performed in triplicate. A one-way analysis of variance (ANOVA) was conducted to compare the results statistically. The data was statistically analyzed using the OriginPro 2023b software. The following discussion presents the findings obtained from 288 h of continuous experimentation on using biological methods to reduce the harmful effects of flue gas and remediate wastewater in a 20 L reactor.

## Results and discussion

### Bacterial consortia characteristics

**Figure 2:**
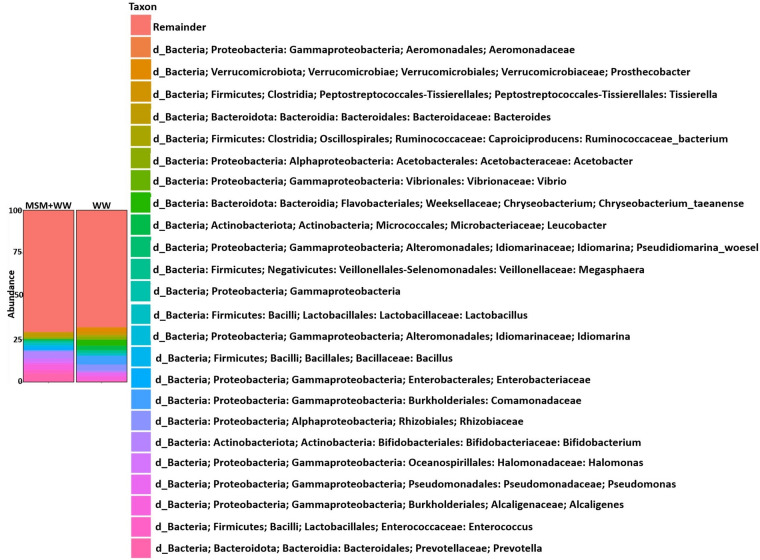
The bar plot summary of the dominant bacterial species in the MSM+WW and WW media biomass.

**Figure 3:**
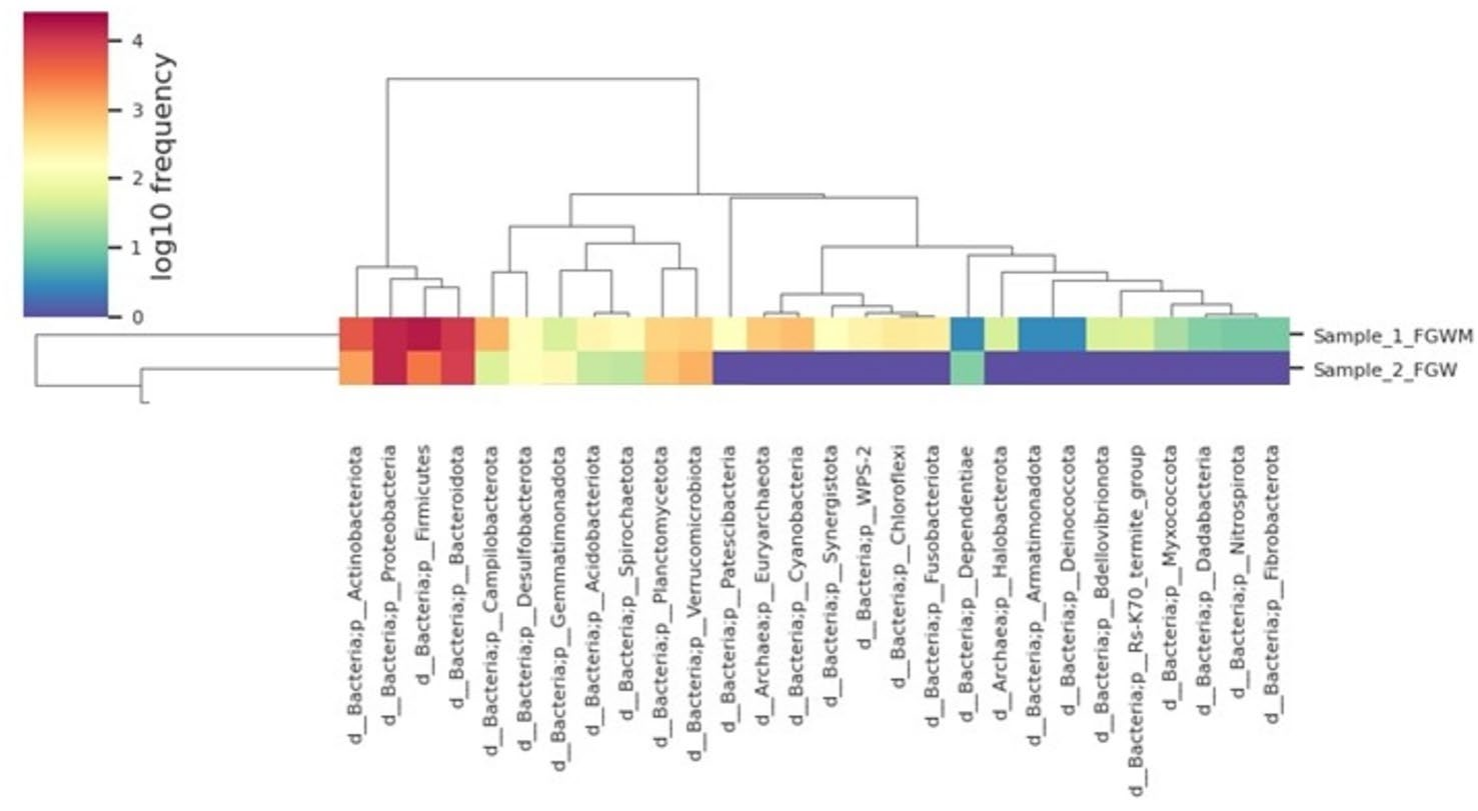
The heatmap comparison of the bacterial species obtained from both columns.

The suspended medium was analyzed using genetic sequencing to study the bacterial communities. This study employed MiSeq high-throughput sequencing to examine bacterial diversity comprehensively. Figure 2 illustrates bacterial distribution at various taxonomic levels, including species, phylum, order, genus, family, and class. In addition, Fig. 3 shows the comparison of species richness in both media through the use of heatmaps. Figure 3 illustrates the disparity in bacterial species diversity between the MSM+WW and WW media. The color gradient from green to red indicates the bacteria concentration, while purple signifies the bacteria's absence. The heat maps display the prevalence of bacteria in the MSM+WW media. In contrast, the WW medium lacks over 50% of the bacteria, indicating a deficiency in bacterial diversity.

Both mediums displayed a significant range of microorganisms, which could be distinguished based on observable traits. The prevailing microorganisms in both media consisted mainly of uncultured bacteria, although a few identified species were present, including *Chryseobacterium taeanense*, *Flavobacterium sasangense*, *Lactobacillus ruminis*, glacier bacterium, and *Synechocystis*. *Prevotella* was the most prevalent bacterial genus in the MSM+WW medium, making up 5.08% of the total. In contrast, *Chryseobacterium* was the most abundant genus in the WW media, accounting for 3.51%. The other notable bacterial genera present in both samples included *Rhizobiaceae*, *Leucobacter*, *Alcaligenes*, *Enterococcus*, *Halomonas*, *Pseudomonas*, *Bacillus*, *Lactobacillus*, *Bifidobacterium*, *Bacteroides*, and so on. The bacteria were categorized into various taxonomic groups such as Bacteroida, Gammaproteobacteria, Bacilli, Clostridia, Actinobacteria, Alphaproteobacteria, etc. The predominant bacterial phyla were Proteobacteria (46.08%), Bacteroidata (30.44%), Actinobacteriota (10.54%), and Firmicutes (10.29%).

All the bacteria displayed both halophilic and thermophilic traits. The media comprised a mixture of gram-positive and gram-negative bacteria, encompassing obligate aerobes and facultative anaerobes. The metagenomics analysis ruled out the existence of algae, fungi, and yeast, leading to the isolation of exclusively uncontaminated bacterial strains. The prevailing bacterial genus in the reactor was primarily composed of sulfur-oxidizing bacteria, *Prevotella* and *Chryseobacterium,* which had a substantial impact in efficiently eradicating SO_2_^31^. Extensive research has been conducted on the Pseudomonas and Bacillus genera because of their proven ability to remove nitrates and NOx effectively, leading to efficient nitrogen removal^32^. *Enterococcus*, *Halomonas*, *Prevotella*, *Lactobacillus*, and *Bacillus* have been proven to be proficient carbon-utilizing bacteria^33–35^.

### Flue gas (CO_2_, SO_2,_ and NO) bio-mitigation and biomass productivity

**Figure 4:**
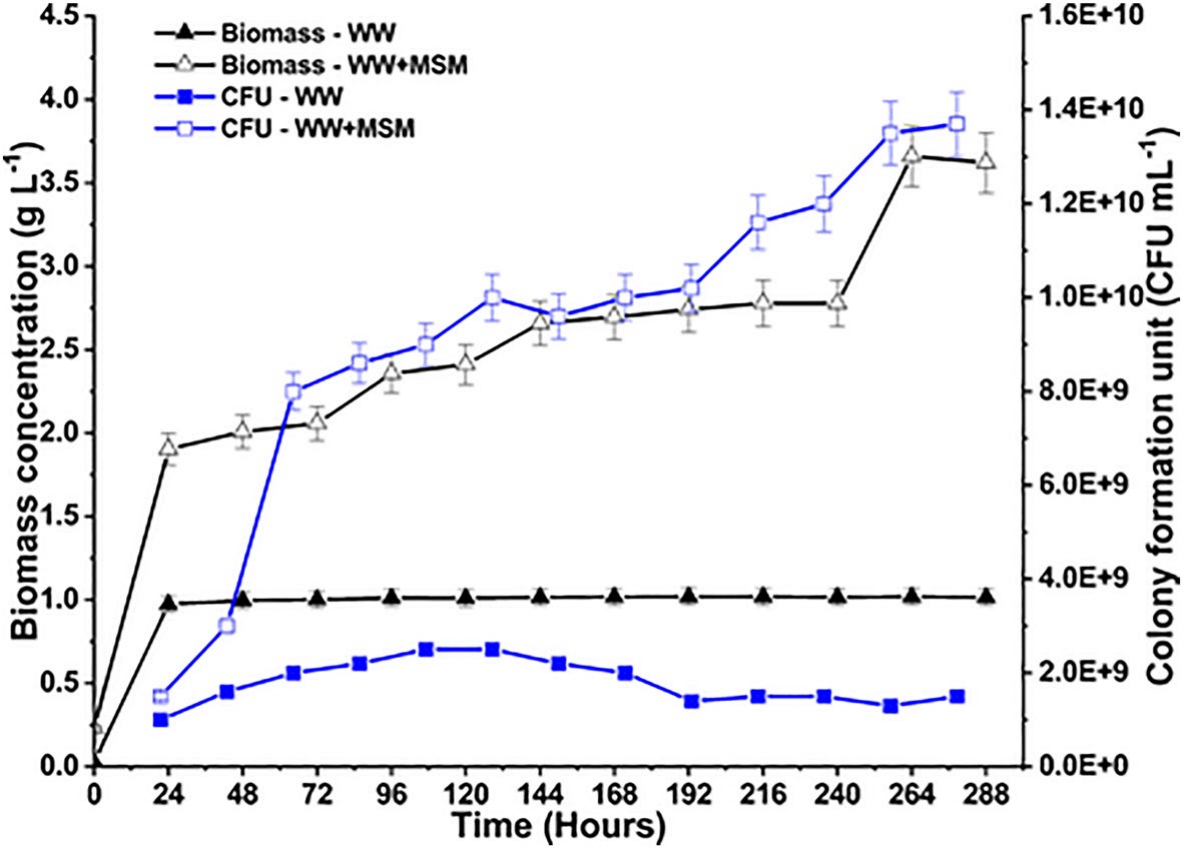
The growth phase analysis by biomass concentration and CFU measurement throughout the cultivation period.

The bacterial consortia growth is depicted as a function of biomass concentration, and CFU in Fig. 4. The OD-dry weight curve obtained for both media is shown in Supplementary Figs. S1 and S2. No lag phase was observed in either medium (MSM+WW and only WW) as the bacteria were acclimatized to the nutritional media and supplemented with flue gas to shorten the lag phase. At the onset of the cultivation, the bacteria entered the log phase and proliferated rapidly. The bacteria utilized the medium’s maximum available substrate and nutrients for growth. The bacteria best thrived in the MSM+WW-based media due to the availability of more substrate in the system, thus giving higher growth values. The flue gas fed to the system continuously provided growth substrate in the form of carbon, nitrogen, and sulfur in addition to the nutrients available through the WW. In the culture media with only WW, the bacteria entered a stationary phase after 48 h, in which growth was extremely slow or constant. After 72 h, an exponential decay occurred as bacteria in the culture couldn’t multiply or thrive due to the unavailability of nutrients. Without MSM, the WW media had limited substrate available, and by the end of 72 h, the bacteria had used up all the nutrients in the medium. Although the death phase started at 72 h, the experiment was continued up to 288 h to maintain uniformity in representing the results of both (WW and MSM+WW) media experiments.

In the MSM+WW media, the bacteria stayed in the exponential growth phase till the end of the cultivation period due to the abundant availability of nutrients. The highest biomass obtained was 3.66 and 1.02 g L^−1^ for MSM+WW and WW media, respectively. The biomass productivity values were calculated using the values of dry-weight biomass. Due to the lack of MSM in the WW media, the bacteria could not flourish even after being continually fed with the flue gas. Therefore, it is evident that, in addition to the growth substrate (flue gas+WW), a minimal salt medium is required for the proper growth of bacteria. Minimal media can replicate precise environmental conditions from which the bacteria is originally derived. The NaCl provides salinity to the media, which is vital for the bacterial consortia as these were obtained from a hypersaline environment. A sufficient amount of salt in the MSM+WW media indicated that the bacteria grew better in that particular environment. During bacterial growth, every carbon, hydrogen, and oxygen molecule creates new cells or yields products. Hence, bacterial growth is subjected to the rule of conservation of matter, irrespective of the intracellular processes involved^36^.

The CFU analysis was performed to distinguish between dead and living biomass in the culture. CFU plating considers just the living bacteria that contribute to flue gas reductions. The CFU analysis demonstrated that viable cells decreased over time from 2.5 × 10^9^ to 1.3 × 10^9^ CFU mL^−1^ for WW media. The nutrients became scarce at the end of cultivation time, and the cells’ ability to take up substrate decreased. The highest colony of bacteria cultivated in the system fed with MSM+WW media, where they received the maximum amount of substrate and thriving conditions to reproduce more. The cells kept increasing till the end of the cultivation period up to 1.37 × 10^10^ CFU mL^−1^. Hence, the bacterial biomass and growth findings suggested that the bacterial consortia can adapt well to the flue gas and WW emitted from industries for their growth.

**Figure 5:**
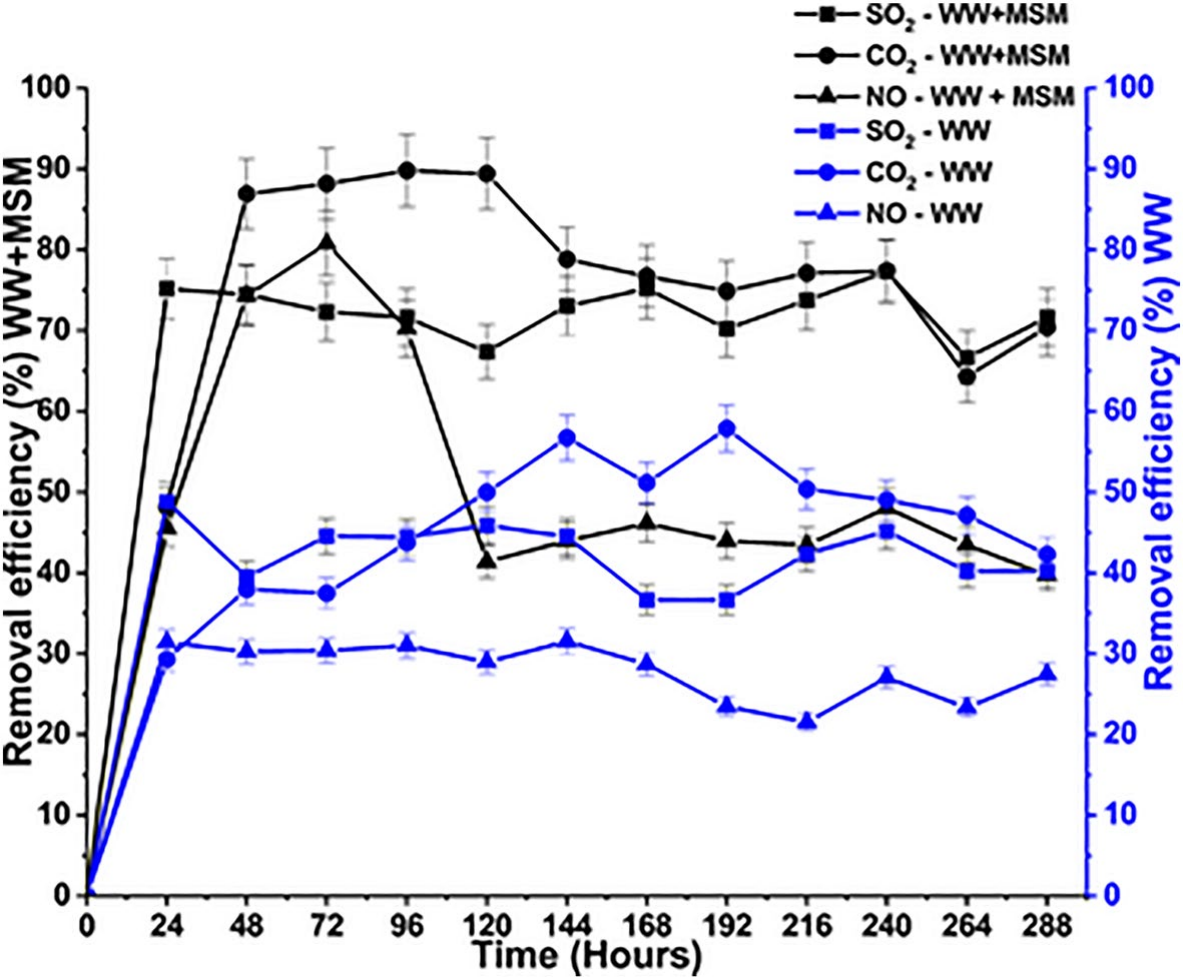
Removal efficiency in the MSM+WW and WW media during the cultivation period.

The bio-mitigation of CO_2_, SO_2_, and NO in the bubble column reactor by bacterial consortia was the primary focus of the reactor investigation. The carbon, nitrogen, and sulfur sources given solely in gaseous form by the flue gas mixture served as the substrate for microbial development. The initial hours were allocated to dissolving flue gas in the media to create equilibrium. The growth commenced once the flue gas concentrations in the gaseous and liquid phases were balanced, and the mitigation of flue gases started. The CO_2_ (g) dissociates to HCO_3_^−^, which is used by bacteria as a carbon substrate for growth, thereby reducing CO_2_ (g) emissions^37^. The NO can be converted to NO_3_^−^ and NO_2_^−^ by nitrification. In the liquid phase, nitrate-reducing bacteria (NRB) can reduce NO_3_^−^ to NO_2_^−^. Hence, the NRB uses NO_3_^−^ and NO_2_^−^ as nitrogen substrates, thus reducing the NO (g) emissions^38^. Similarly, Given the high solubility of SO_2_ in liquids, SO_2_ dissolves in water to form SO_3_^2−^ and SO_4_^2−^ under aerobic conditions. Hence, the sulfate-reducing bacteria (SRB) reduce SO_2_ to be used as a sulfur source^39^. The highest removal rate achieved with MSM + WW media was 89.80, 77.30, and 80.77% for CO_2_, SO_2_, and NO, respectively. The highest removal achieved with WW media was 57.88, 45.89, and 31.54% for CO_2_, SO_2_, and NO, respectively. The removal efficiency data for the entire cultivation period is represented in Fig. 5.

The removal efficiency of SO_2_ always remained on the higher side due to its high solubility in aqueous medium. The higher solubility ensured faster availability of SO_2_ for reduction by the SRB. A higher CO_2_ removal rate is achieved as it can cross cell membranes and thus directly enter the cell through diffusion in bacteria^40^. NO (g) has almost negligible solubility in the aqueous media among the gaseous mixture. Bacteria take up soluble NO or NO_3_^-^ as the nitrogen source. However, NO removal is enhanced in the presence of O_2_^41^. Although the system is aerobic and has sufficient dissolved O_2_, NO removal dropped after 96 h in both cases due to the bacteria' excessive use of the dissolved O_2_ and the oxidation reactions in the media. In addition, a stable nitrate removal efficiency was difficult to attain because the reducing activity of the denitrifying bacteria was hampered by excess sulfide accumulation, as they may prioritize the use of sulfide over nitrate, as shown in Fig. 8^42^.

The parametric analysis done for the bio-mitigation of the flue gas experiment is represented in Table 2. The highest biomass productivity in the MSM+WW system is 0.10 g L^−1^ h^−1^, with a specific growth rate of 0.12 h^−1^. Similarly, for the WW system, the biomass productivity is 0.04 g L^−1^ h^−1^, with a specific growth rate of 0.02 h^−1^. The growth of the bacteria and simultaneous biomass production was higher in the system with MSM+WW due to the additional availability of nutrients. The inlet loading of the individual gas in the gaseous mixture was 624, 0.85, and 4.86 g L^−1^ h^−1^ for both systems for CO_2_, SO_2_, and NO, respectively. Hence, the elimination capacity obtained for the MSM+WW system was 550.20, 0.65, and 3.78 and 301.60, 0.34, and 1.28 g L^−1^ h^−1^ for the WW system for CO_2_, SO_2_, and NO, respectively. The elimination capacity of the MSM+WW system was higher than that of the WW system due to the higher growth of bacteria and greater utilization of nutrients. The actual gas utilization efficiency calculated for the feed of each gas was 44.50, 37.40, and 24.10% in the MSM + WW system and 24.12, 22.30, and 15.70% in the WW system for CO_2_, SO_2,_ and NO, respectively. Similarly, the bio-fixation rate concerning the biomass formed was calculated as 0.035, 0.009, and 0.005 g L^−1^ h^−1^ for the MSM+WW system and 0.001, 0.0003 and 0.0004 g L^−1^ h^−1^ for WW system for CO_2_, SO_2,_ and NO, respectively.

**Table 2:** Parametric analysis of the bio-mitigation process for 288 h of cultivation period.

Parameters	Minimal salt media + Wastewater			Wastewater		
	CO_2_	SO_2_	NO	CO_2_	SO_2_	NO
Highest removal efficiency (%)	89.80	77.30	80.77	57.88	45.89	31.54
Biomass productivity (g L^−1^ h^−1^)	0.10			0.04		
Specific growth rate (h^−1^)	0.12			0.02		
Biomass yield (g cells produced/g C/S/N consumed)	0.0016	0.16	0.08	0.0009	0.095	0.044
Inlet loading (g L^−1^ h^−1^)	624	0.85	4.68	624	0.85	4.68
Elimination capacity (g L^−1^ h^−1^)	550.20	0.65	3.78	301.60	0.34	1.28
Residence time (min)	10			10		
Bio fixation rate (g L^−1^ h^−1^)	0.035	0.009	0.005	0.001	0.0003	0.0004
Actual gas utilization efficiency (%)	44.50	37.40	24.10	24.12	22.30	15.70

Table 3 compares the results obtained in the current investigation for reactors with varying volumes, microorganisms, cultivation days, and flue gas concentrations. The reported studies may have obtained a lower biomass concentration as a result of having a small reactor volume, a shorter cultivation period, and using only one bacterial species. The present study is a novel approach to simultaneously treating flue gas (CO_2_, SO_2,_ and NO). The present work may have achieved a higher removal efficiency due to the bioreactor, which involved fewer mechanical parts within the reactor and thus provided more area for diffusion and mass transfer^43^. The mixing in the reactor was dependent solely on the bubbling velocity of the gas, providing a superior diffusion mechanism and supporting higher cell density compared to CSTR and packed bed reactors^44–46^. The reactor design achieved a residence time of 10 min which increased the flue gas diffusion time and the dissolved substrate’s and bacteria’s reaction time in the media. Since the bacterial culture had additional time to react, it could digest the substrate more efficiently and produce more biomass than other reactor systems.

**Table 3:** Comparison of the present study with reported literature for CO_2_, SO_2,_ and NO bio-mitigation.

Reactor system	Inlet concentration in gas (v/v%)	Cultivation days (h)	Removal efficiency (%)	Biomass production (g L^−1^)	Species	References
Biotrickling filter	NO—0.005–0.04	1200	94.2	–	*Pseudomonas putida*	Jiang et al.^47^
Biofilter	NO—0.3	40	65	–	Denitrifying and Fe(III) reducing bacteria	Zhang et al.^48^
Biofilter	NO—0.06 and SO_2_—0.018	100	98.08 and 99.61	–	NRB and SRB	Sun et al.^39^
CSTR	CO_2_—5	60	75	–	*Bacillus* sp.	Sundaram et al.^34^
Integrated bioreactor	SO_2_—0.004	180	85	2.7 × 10^11^ CFUs g^−1^	*Paenibacillus* sp*.* and *Ralstonia* sp.	Lin et al.^49^
Lab scale bioreactor	NO—0.075 and CO_2_—10	144	93.44 and 86.50	4.95	*Bacillus tropicus* and *Bacillus cereus*	Anand et al.^12^
Flask batch	CO_2_—15	144	98	1.12	*Halomonas stevensii*	Mishra et al.^50^
Thermophilic biofilter	SO_2_—0.0075	6000	93.10	9.45 × 10^6^ CFU mL^−1^	Desulfurizing bacteria	Zhang et al.^48^
Biotrickling tower	NO—0.16 and SO_2_—0.11	480	80.85 and 100	–	Desulfurizing and Denitrifying bacteria	Wang et al.^51^
Biofilter	NO—1.63, SO_2_—0.6 and CO_2_—15.5	168	82.81, 100 and 75.23		Bacterial consortia	Xie et al.^52^
Bubble column bioreactor	NO—0.075, SO_2_—0.0141 and CO_2_—10	288	89.80, 77.30 and 80.77	3.66	Bacterial consortia	Present study

### Analytical assessment of DO, BOD5, COD, pH and salinity

**Figure 6:**
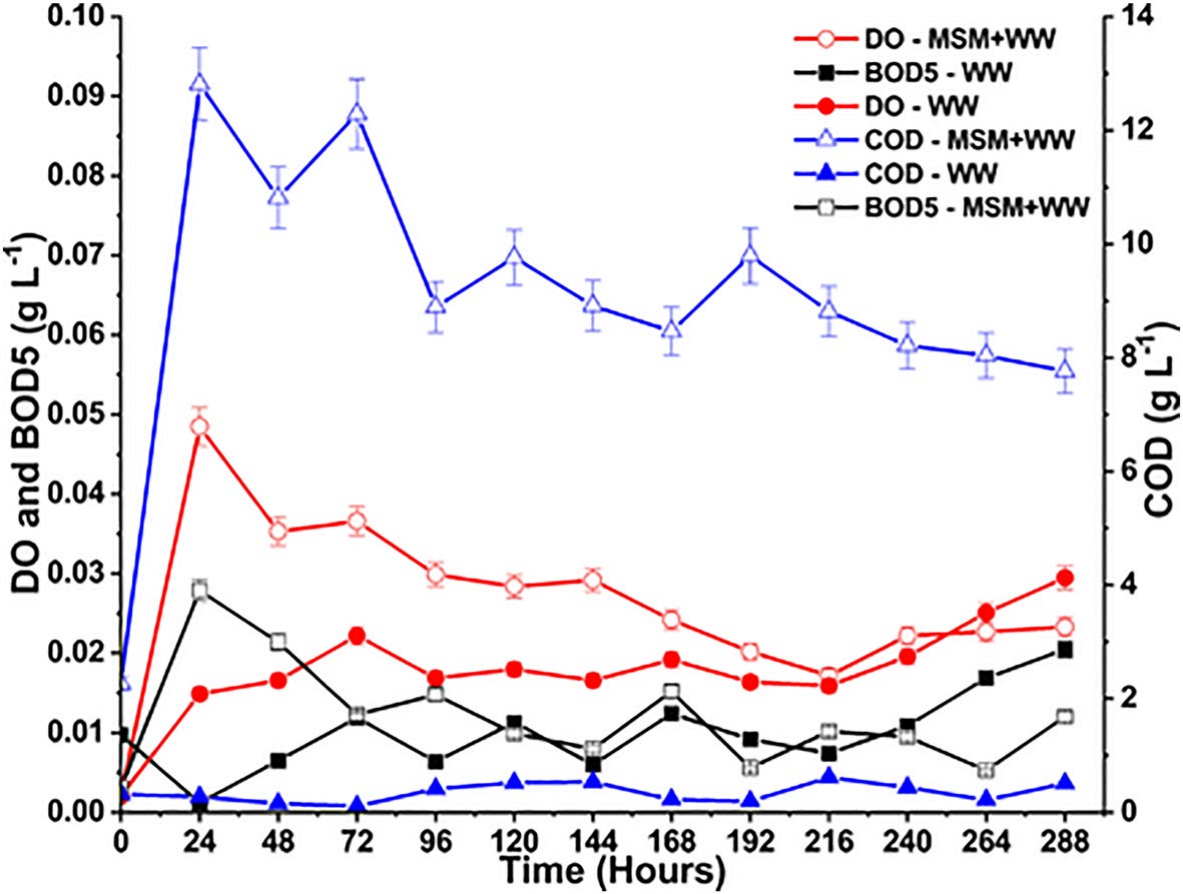
The DO, BOD5, and COD variation of bio-mitigation of flue gas with MSM+WW and WW.

COD and BOD are two common approaches to categorizing organic materials in an aqueous medium. High organic content in wastewater is known to cause several issues, such as turbidity, DO depletion, and disruption of bacterial culture’s normal functioning^53^. During the cultivation period, DO, BOD, and COD were crucial factors, and Fig. 6 depicts their variation in the aqueous medium. In the present study, MSM+WW and WW media contained up to 0.048 and 0.03 g L^−1^ of DO, respectively, which proved to be an aerobic environment where the bacteria thrived more effectively^53^. Literature suggests that 0.009 g L^−1^ of DO is the absolute minimum concentration for bacteria to survive in an aerobic environment^53,54^. Therefore, it was clear that DO levels during cultivation were consistently higher than the DO threshold. The trend in the DO levels showed that the DO was being continuously depleted and replenished in both media till the end of the cultivation period. The DO depletion was insignificant in the WW media as the biological activity was lesser in this media compared to the MSM+WW media. DO is the fuel for most cellular processes because bacteria synthesize Adenosine triphosphate (ATP) and the oxidizing reactions involving CO_2_, SO_2,_ and NO to convert to CO_3_^2−^, HCO_3_^−^, SO_3_^2−^, SO_4_^2−^, NO_3_^−^, and NO_2_^−^^39,55^.

The oxygen required for aerobic digestion by the bacteria in the media is known as its BOD. This mechanism degenerates the complex organic material and breaks it into simpler molecules, increasing the oxygen tension in water and leading to a rise in BOD^56,57^. The BOD values in both media systems are below 0.008 g L^−1^, the highest limit for the criteria of severely contaminated water^58^. COD quantifies the media's susceptibility to oxidation of both organic and inorganic materials, while BOD refers primarily to the biological oxidation of organic matter. The BOD test uses bacteria, while the COD test uses a chemical oxidizing agent to oxidize the organic content in the sample. The COD test is frequently used in conjunction with the BOD test to assess the amount of organic material in the media that is not biodegradable^59,60^. In the present study, the COD level of the MSM+WW media ranged between 12 and 8 g L^−1^, indicating that bacteria in this medium did not digest most organic or inorganic material. It was significantly higher than that of the WW media. In the WW media, the COD level was much lower, with values between 0.034 and 0.5 g L^−1^. Biodegradable organics have a COD between 1.33 and 1.50 times the BOD, and in both cases, the COD: BOD ratio was above 2. Since biodegradable matter is consumed during bio-mitigation and, non-biodegradable organics are left to yield comparatively higher COD values. The impacts of microbial end products, such as organic acids and alcohols produced during the process, further amplify the rise in the COD: BOD ratio^61,62^.

**Figure 7:**
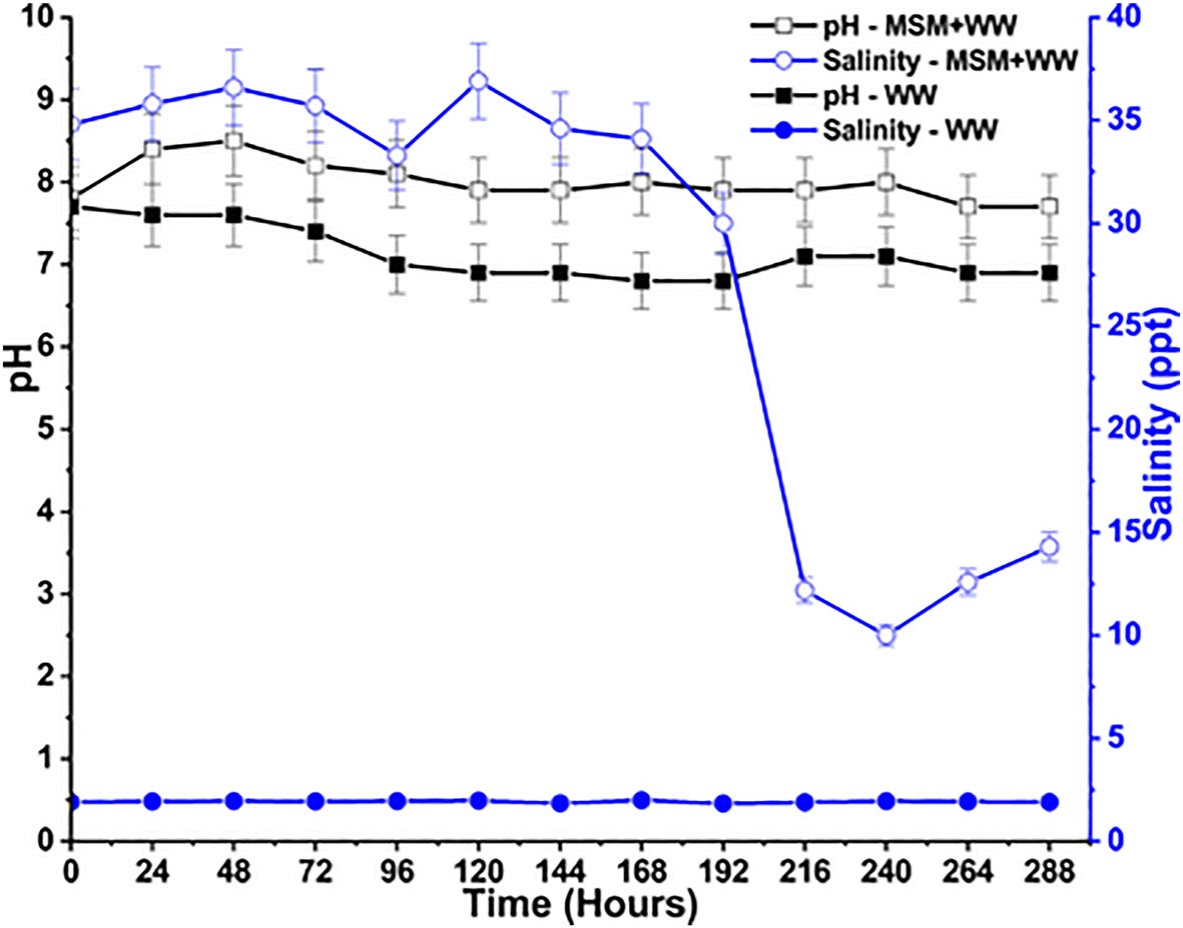
The pH and salinity variation of bio-mitigation experiment with MSM+WW and WW.

The variation of salinity and pH throughout the cultivation period from inoculation to the last day is represented in Fig. 7. The addition of a significant quantity of MSM to the MSM+WW media played a vital role in determining the salinity of the system. Meanwhile, the WW media did not contain any components that contributed to the salinity. The mixed bacterial culture obtained from SSL thrives at a higher salinity level of ≤ 70 ppt^63^. The bacterial culture exposed to a greater salinity gradient would have their cells burst under the osmotic pressure and die off quickly. Also, a media with insufficient salinity would not favor the growth of the bacteria^64^. The contribution of salinity in the medium was entirely due to the addition of MSM. Hence, the salinity of the MSM+WW media varied between 10 and 38 ppt, whereas the salinity in the WW media was almost negligible at 1.9 ppt. The bacterial consortia grew adequately in the saline MSM+WW medium; however, the WW medium had insufficient biomass. This revealed that MSM is required to give growth factors to culture conditions to promote bacterial multiplication^64^. The salinity was stabilized in the MSM+WW media until 168 h as the bacteria grew; however, it decreased drastically after that due to excessive biomass accumulation.

The pH was observed between 7 and 8.9 for both reactor mediums during bio-mitigation. The reactor’s pH remained neutral to enhance biomass accumulation^65^. When CO_2_ (g) dissolved, it reacted in the aqueous medium to form weak carbonic acid (H_2_CO_3_). Similarly, NO dissolves in water to form nitric (HNO_3_) and nitrous acid (HNO_2_), and SO_2_ dissolves to form sulphuric acid (H_2_SO_4_), which are initially responsible for lowering the reactor’s pH levels^39^. The biomass formation in the reactor balanced the pH of the medium, as biomass accumulation made the medium basic. These shifts in value could also be attributed to the media's alkalinity, which increased and decreased due to inorganic reactions in the media. The pH level of the MSM + WW media was slightly higher due to the higher biomass and inorganic matter accumulation rate than in the WW media. No pH buffers were added to the media externally to maintain the pH in the system.

### Total nutrient assimilation and balance (carbon, nitrogen, and sulfur)

**Figure 8:**
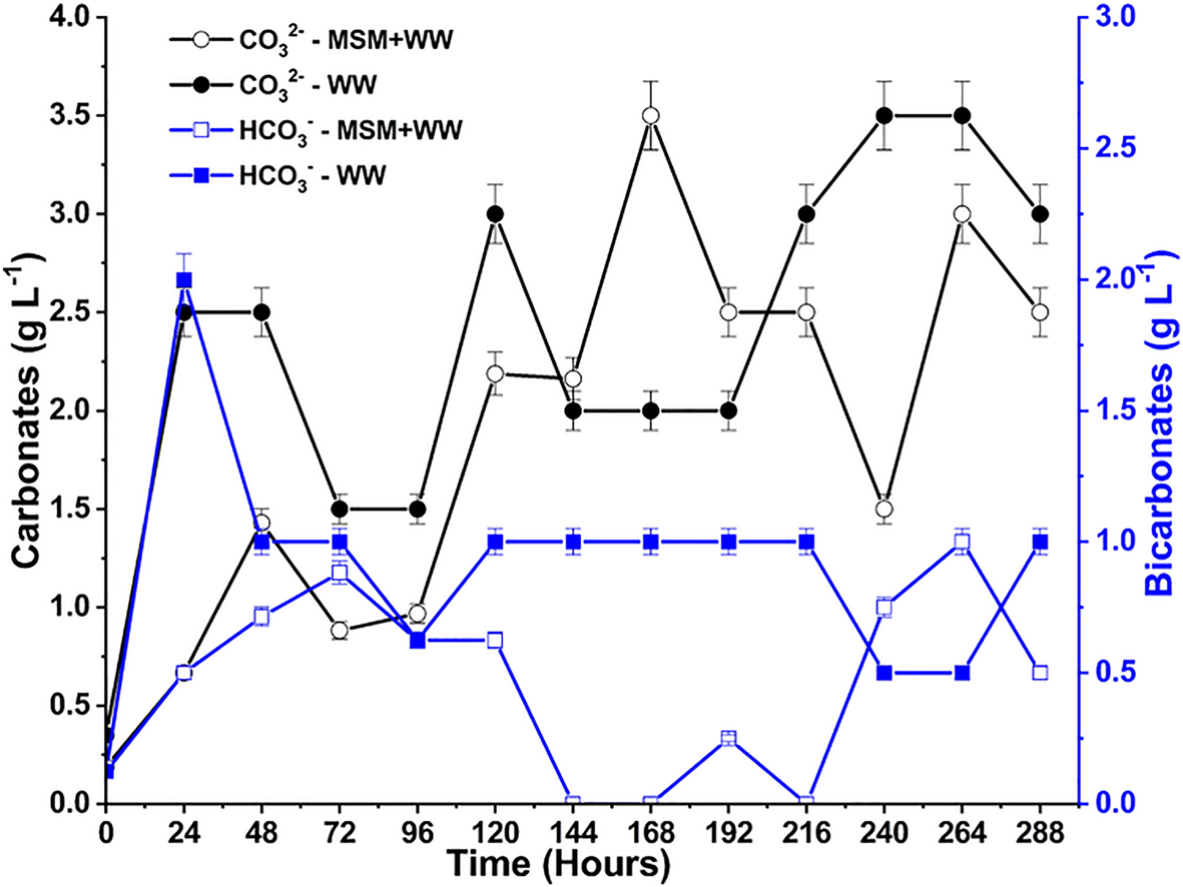
The CO_3_^2−^ and HCO_3_^2−^ variation during bio-mitigation with MSM+WW and WW.

The CO_2_, SO_2_, and NO dissolved in the medium undergo dissociation to form inorganic compounds, which are then utilized by the bacteria as substrates. The dissolved CO_2_ produced H_2_CO_3_ in the media via a hydration reaction (Eq. 7). This H_2_CO_3_ dissociated into CO_3_^2−^ and HCO^3−^ ions (Eqs. 8, 9)^39^. These inorganic substances are digested and used as substrates for bacterial growth. The CO_3_^2−^ and HCO^3−^ concentrations vary broadly throughout the cultivation period and are represented in Fig. 8. The bacteria preferentially take in and store HCO_3_^−^ in their cytoplasm as the inorganic carbon source. This meant that HCO_3_^−^ in the media was constantly depleted to the point where the ions couldn’t be detected in the sample on some days. Since H_2_CO_3_ dissociation into HCO_3_^−^ is a very fast reaction, HCO_3_^−^ species were constantly being formed in the media. Thus, there was a constant supply of HCO_3_^−^ for bacterial development. The HCO_3_^−^ concentration in the WW medium was somewhat stable due to non-consumption and lesser growth of bacteria.

7 $$CO_{{2\left( {aq} \right)}} + H_{2} O \Leftrightarrow H_{2} CO_{3}$$

8 $$H_{2} CO_{3} { } \Leftrightarrow HCO_{3}^{ - } + H^{ + }$$

9 $$HCO_{3}^{ - } { } \Leftrightarrow CO_{3}^{2 - } + H^{ + } { }$$

**Figure 9:**
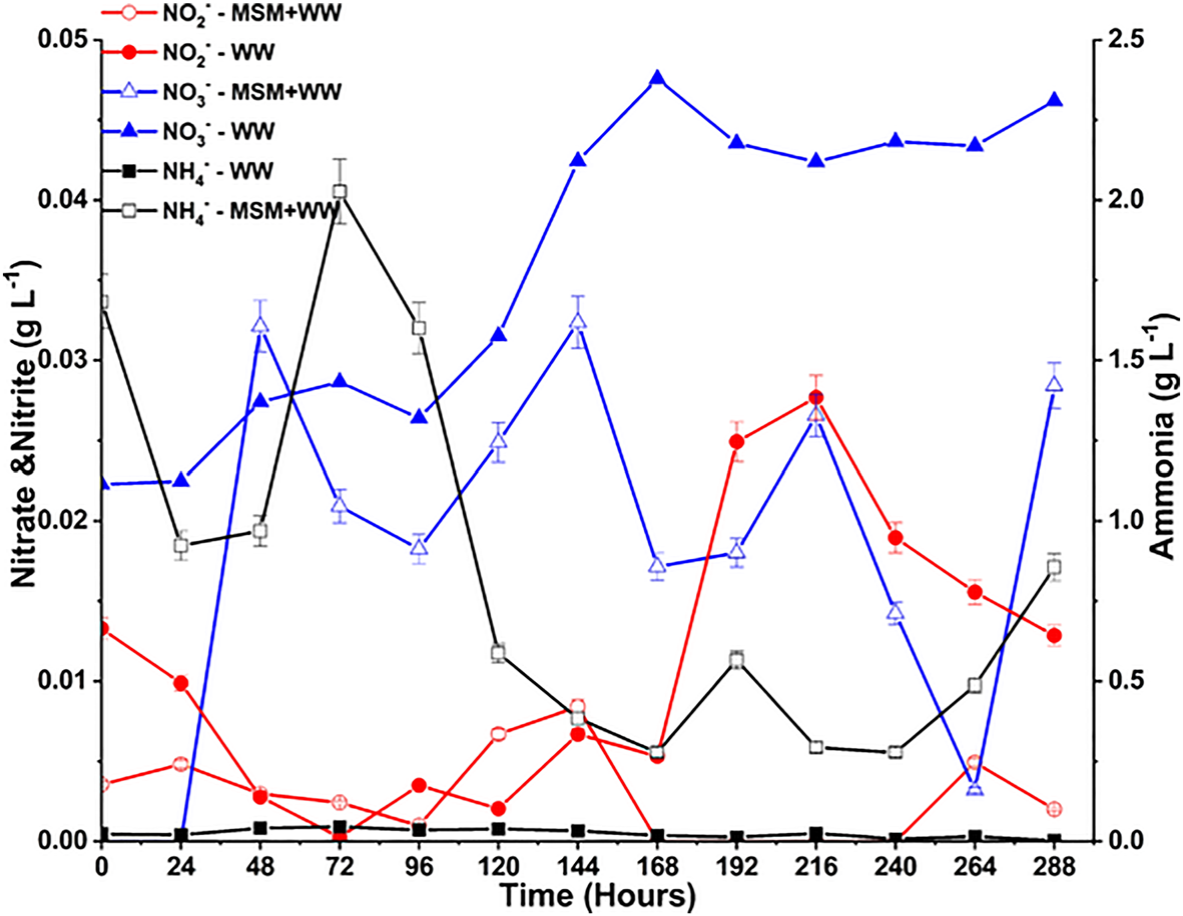
The NO_3_^−^, NO_2_^−^, and NH_4_^+^ variation during bio-mitigation with MSM+WW and WW.

At a pH higher than 6, the NO dissolves in the aqueous medium to form HNO_3_ and HNO_2_. Further, HNO_3_ and HNO_2_ dissociate to form NO_2_^−^ and NO_3_^−^ ions (Eqs. 10, 11)^39^. The heterotrophic nitrifying bacteria use the NO_2_^−^ and NO_3_^−^ ions as the inorganic nitrogen source for their growth. Another nitrogen source provided in the media was through the MSM by adding NH_4_Cl, which dissociated in the media to give NH4^+^. The NH4^+^ is oxidized by the nitrification process in which Nitrosomonas oxidize NH_4_^+^ into NO_2_^−^, and Nitrobacter oxidizes NO_2_^−^ further into NO_3_^−^^66,67^. Bacteria can take up NH_4_^+^, NO_2_^−^, and NO_3_^−^, all three or selectively one, as the nitrogen growth substrate depicted in Fig. 9. The initial NH_4_^+^ concentration is high in the MSM + WW due to the abundant supply of NH_4_Cl, and later, it drops due to the consumption and conversion to NO_2_^−^ and NO_3_^−^. The NH_4_^+^ concentration is negligible in the WW media due to the absence of MSM. The NO_3_^−^ concentration is higher in both the media than the NO_2_^−^, as the NO_2_^−^ is also being continuously oxidized to NO_3_^−^. The NO_2_^−^ and NO_3_^−^ concentrations are higher in the WW media due to the formation of ions and the lower consumption of bacteria. Between NO_2_^−^ and NO_3_^−^, NO_3_^−^ is a more favored substrate for the bacteria as excessive NO_2_^−^ accumulation can be toxic and has inhibitory effects on the bacterial cell^68^.

10 $$HNO_{3} \to ^{ } NO_{3}^{ - } + H^{ + }$$

11 $$HNO_{2} \to ^{ } NO_{2}^{ - } + H^{ + }$$

**Figure 10:**
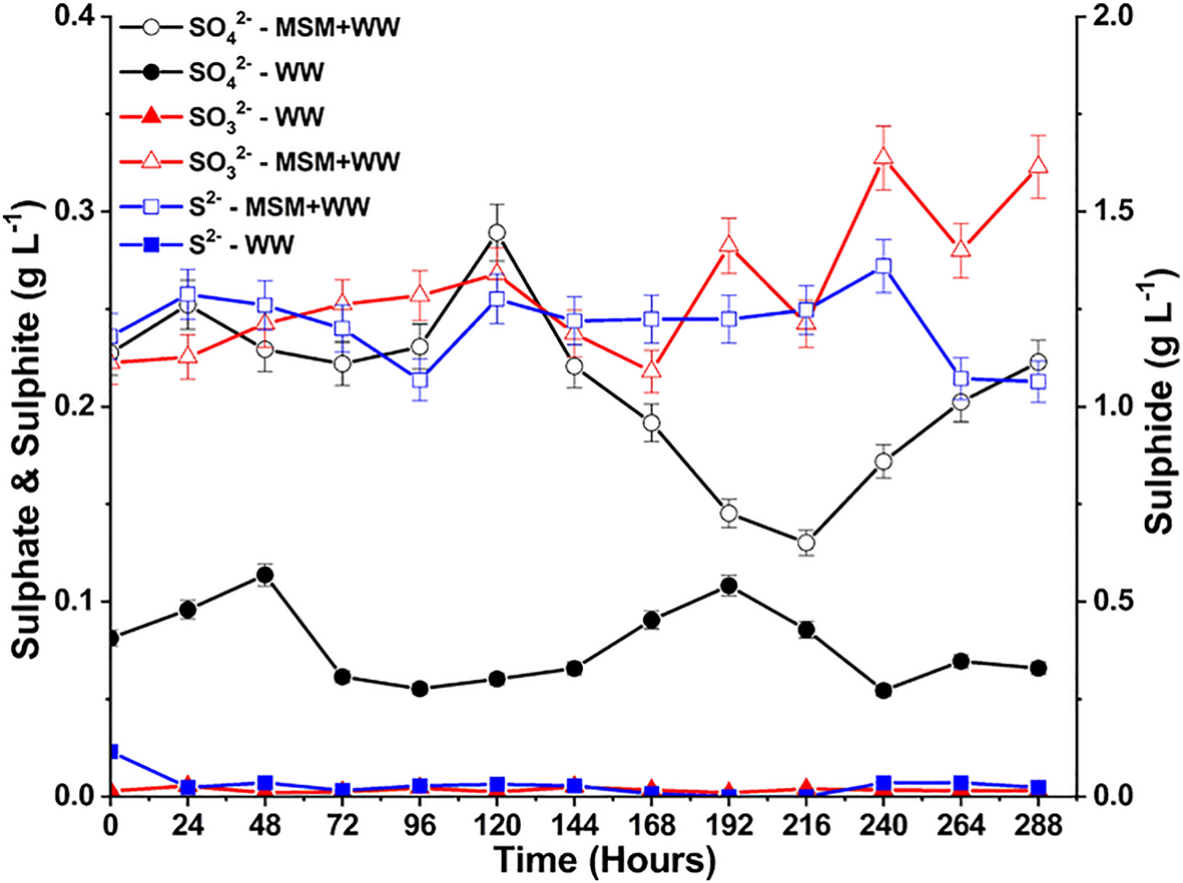
The SO_3_^2−^, SO_4_^2−^ and S^2−^ variation during bio-mitigation with MSM+WW and WW.

Due to the high solubility of SO_2_, it dissolved in the aqueous medium to form sulfurous acid (H_2_SO_3_). Further, H_2_SO_3_ ionizes to form SO_3_^2−^ and bisulfite (HSO_3_^−^) ions (Eqs. 12, 13). These SO_3_^2−^ ions are further oxidized to form SO_4_^2−^ ions in the pH range of 6.5–9 (Eq. 14)^31^. Sulfate-reducing bacteria (SRB) accept sulfate as a terminal electron to degrade organic compounds at the expense of producing S^2−^ ions^69^. The formation of SO_4_^2−^, SO_3_^2−^, and S^2−^ ions in the MSM+WW and WW media is represented in Fig. 10. The formation of the species is higher in the MSM+WW than in the WW media due to the higher activity of bacteria. Due to a greater consumption rate, the SO_4_^2−^ concentration in the MSM+WW media gradually dropped. Due to the constant generation of the ions without further use, S_2_^−^ buildup was likewise high in this medium. Without microbes to consume it, SO_4_^2−^ accumulated in the WW medium. As a result of less SO_4_^2−^ consumption, S_2_^−^ generation was almost negligible. Since SO_3_^2−^ was constantly oxidized to SO_4_^2−^, its concentration in the medium was also low.

12 $$H_{2} SO_{3} + H_{2} O \Leftrightarrow H_{3} O^{ + } + HSO_{3}^{ - }$$

13 $$HSO_{3}^{ - } + H_{3} O^{ + } \Leftrightarrow H_{3} O^{ + } + SO_{3}^{ - }$$

14 $$SO_{3}^{ - } + SO_{3}^{2 - } + O_{2} \to 2SO_{4}^{2 - }$$

**Figure 11:**
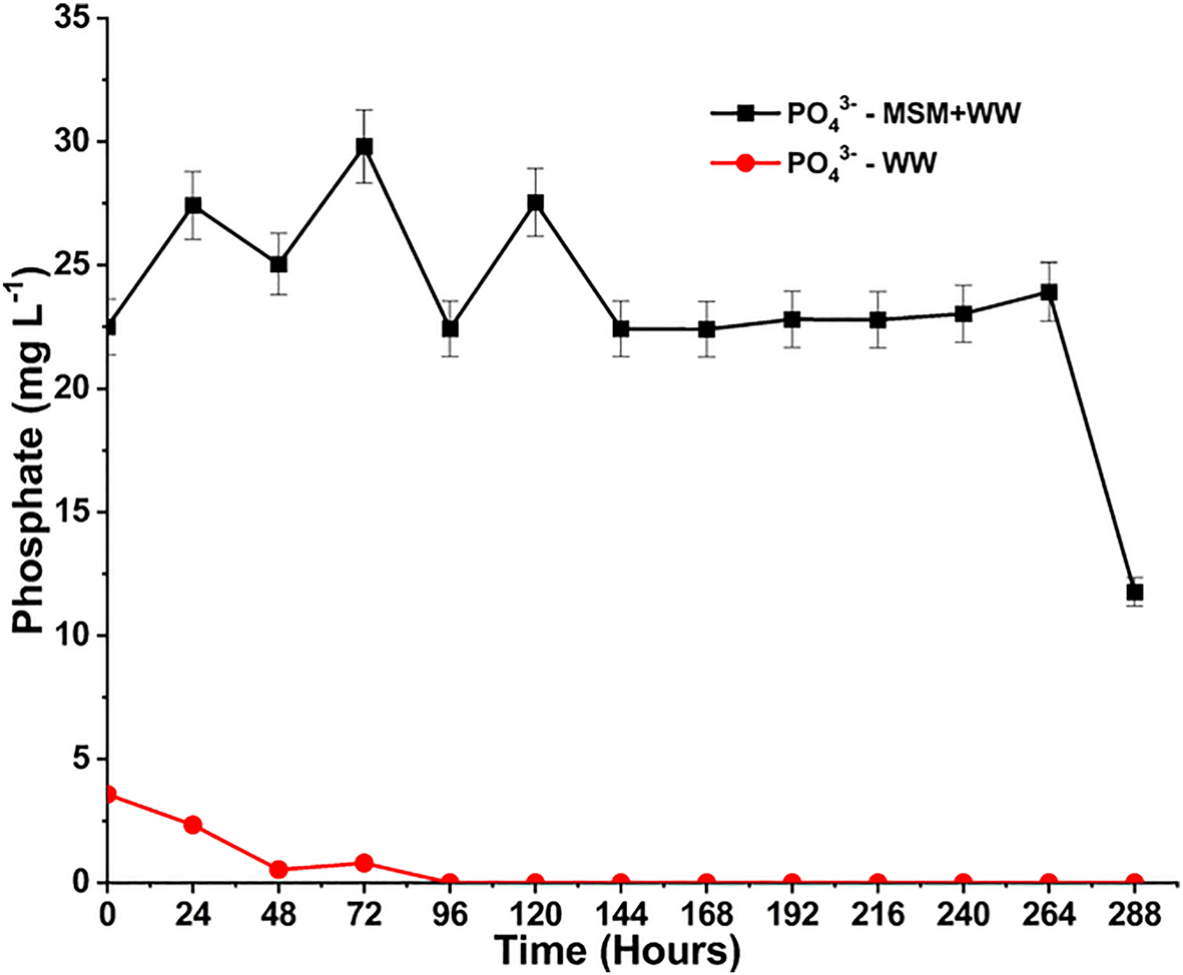
The PO_4_^3−^ variation during bio-mitigation with MSM+WW and WW.

PO_4_^3−^ is one of the essential nutrients for regulating bacterial cell metabolism and is represented in Fig. 11^70^. This nutrient is only supplied to the medium via MSM by adding K_2_HPO_4_. Consequently, we can observe in the MSM+WW media that the use of PO_4_^3−^ fluctuated initially but eventually stabilized and dropped due to the constant depletion. The WW provided a limited amount of PO_4_^3−^ to the media; hence, as time passed, the level of PO_4_^3−^ in the media decreased to nil. The process involves the total utilization of PO_4_^3-^ from wastewater by bacteria in the media, resulting in wastewater remediation. As the inorganic nutrients are continuously formed in the media due to the dissolution of flue gas, it is only possible to measure the efficiency of nutrient utilization over time rather than the efficiency of nutrient removal. Based on the nutrients’ formation and consumption, each nutrient’s average utilization efficiency is estimated by Eq. (3) and depicted in Table 4.

**Table 4:** The maximum nutrient utilization efficiency during the cultivation period.

	Nutrient utilization efficiency (g L^−1^ h^−1^)							
	CO_3_^2−^	HCO_3_^2−^	NO_3_^−^	NO_2_^−^	NH_4_^+^	SO_3_^2−^	SO_4_^2−^	PO_4_^3−^
Minimal salt media + wastewater	0.083	0.026	0.0007	0.0044	0.04	0.005	0.005	0.0004
Wastewater	0.015	0.006	0.0001	0.0004	0.01	0.002	0.0001	0.0001

Carbonate (CO_3_^2−^), bicarbonate (HCO_3_^−^), phosphate (PO_4_^3−^), sulfite (SO_3_^2−^), sulfide (S^2−^), sulfate (SO_4_^2−^), nitrate (NO_3_^−^), nitrite (NO_2_^−^), ammonium (NH_4_^+^).

Based on the estimation of different nutrients, biomass, and gaseous mitigation of the flue gas, the C, N, and S balance of the system was established. The C, N, and S balance is shown in Table 5. Only the flue gas and wastewater supplied to the bioreactor is considered the source of C/N/S in the system. Hence, the total gram moles of carbon, nitrogen, and sulfur provided to the system are 1763.86, 15.46, and 6.41 g, respectively, in both MSM+WW and WW media. The mass of C, N, and S withdrawn in the gaseous form from the inlet gaseous mixture was 784.60, 7.45, and 1.76 g, respectively, for MSM+WW medium and 1092.45, 11.16, and 4.20 g, respectively for WW medium. The C/N/S assimilated in the form of biomass was calculated from the results obtained by the CHNS analysis of the biomass. The CHNS analysis of the WW biomass resulted in (C—2.07%, H—3.31%, N—0.47%, S—0.32%, and O—93.82%), whereas the MSM+WW media biomass resulted in (C—16.90%, H—3.87%, N—3.58%, S—0.37% and O—75.28%). Hence, the C/N/S content in the biomass was 781.15, 4.74, and 2.37 g, respectively, for the MSM + WW medium and 248.13, 0.92, and 0.27 in the WW medium. The C content for the nutrients (CO_3_^2−^, HCO_3_^−^ and dissolved CO_2_) obtained was 174.97 g in MSM+WW media and 340.59 g in WW media. Similarly, the N content for the nutrients (NO_3_^−^, NO_2_^−^, and dissolved NO) were 2.99 and 3.25 g for MSM+WW and WW media, respectively. The S content for the nutrients (SO_4_^2−^, SO_3_^2−^, and dissolved SO_2_) were 2.17 and 1.88 g for MSM+WW and WW media, respectively. Hence, the total C/N/S mitigated in all forms were 1740.72, 15.18, and 6.30 g for MSM+WW medium and 1681.17, 15.33, and 6.35 g for WW media, respectively. Most of the flue gas in the WW just dissolved in the medium without being utilized by the bacteria. The difference in the mass of C/N/S can be attributed to the formation of various products, which is supported by the GC–MS analysis reported in the impending section.

**Table 5:** Total carbon, nitrogen, and sulfur balance in the inlet and outlet of the bioreactor system.

Forms of Carbon (C), Nitrogen (N), and Sulfur (S) in the system	Mass of C/N/S (g) in MSM + WW media	Mass of C/N/S (g) in WW media
Total C input to the system through CO_2_ (g)	1763.86	1763.86
C withdrawn in the gaseous phase as CO_2_ (g)	784.60	1092.45
C assimilated in the form of biomass	781.15	248.13
C present in the form of carbonates	98.68	124
C present in the form of bicarbonates	23.49	24.59
C present in the form of dissolved CO_2_ (g)	52.8	192.0
Total C utilized	1740.72	1681.17
Error (%)	1.31	4.69
Total N input to the system through NO (g)	15.46	15.46
N withdrawn in the gaseous phase as NO (g)	7.45	11.16
N assimilated in the form of biomass	4.74	0.92
N present in the form of nitrate	1.22	1.61
N present in the form of nitrite	0.49	0.79
N present in the form of dissolved NO (g)	1.28	0.85
Total N utilized	15.18	15.33
Error %	1.81	0.90
Total S input to the system through SO_2_ (g)	6.41	6.41
S withdrawn in the gaseous phase as SO_2_ (g)	1.76	4.20
S assimilated in the form of biomass	2.37	0.27
S present in the form of sulphate	1.51	1.22
S present in the form of sulphite	0.13	0.18
S present in the form of dissolved SO_2_ (g)	0.53	0.48
Total S utilized	6.30	6.35
Error %	1.72	0.94

### Product quantification analysis

**Figure 12:**
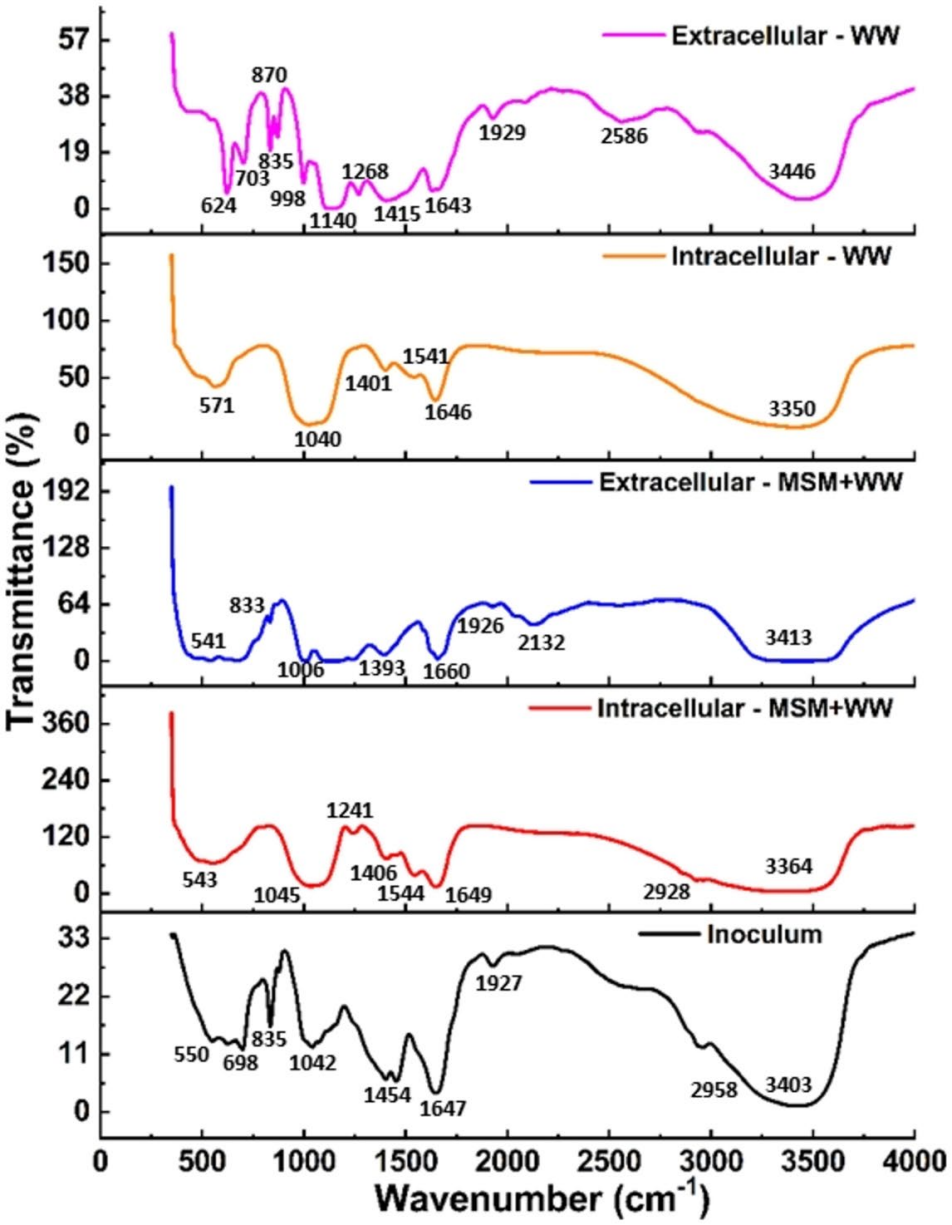
The functional group quantification of IC and EC samples.

Figure 12 shows the characteristic absorption bands determined by analyzing the FT-IR data for the cell lysate and supernatant from the MSM+WW and WW media bioreactor samples. The FT-IR spectra indicated the shifting of various peaks due to the change in media composition. The spectrum peaks obtained were between the mid-IR region’s 3500–400 cm^−1^ range. The broad and strong peak in the 3500–3000 cm^−1^ region implicated the single bond region. It refers to the hydroxyl group (-OH) of carboxylic acid. The wavenumber increased in the case of the EC sample and decreased in the IC sample, signifying an alteration in the H bonding^71^. The peaks in the single bond area between 3000 and 2000 cm^−1^ revealed linear aliphatic compounds and aldehydes undergoing (–CH) stretching. The presence of a peak in the double bond region of wave number 1700–1450 cm^−1^ indicated either the (–C=O) group of carbonate and bicarbonate ion formed due to the CO_2_ (g) fixation or due to the bending of (–CH) present in the aliphatic group and (–OH) bending of simple aromatic compounds^71^. The occurrences of peaks in the region of wave number 1300–1100 cm^−1^ represented the secondary amine CN group and (–C–O) group of proteins. The increase and decrease in the wavenumber indicate the interaction between the nitrogen of the amino group due to the feed flue gas. The C–O–C and C–O stretching of the COOH group from lipids or polysaccharides were confirmed by the peaks obtained in the region of wave number 1100–1000 cm^−1^^72^. The presence of compounds having unsaturated (double bond) and C–H bond of long-chain alkanes was observed by the peaks obtained in the region of wave number 800–600 cm^−1^. The changes in the samples’ wavenumber between 500 and 600 cm^−1^ indicate the shifting of sulfide and amide groups^72^. Thus, the complete FT-IR spectra of cell lysate revealed that the samples collected from the reactor contained a wide variety of functional groups associated with polysaccharides, lipids, and carboxylic acids.

**Figure 13:**
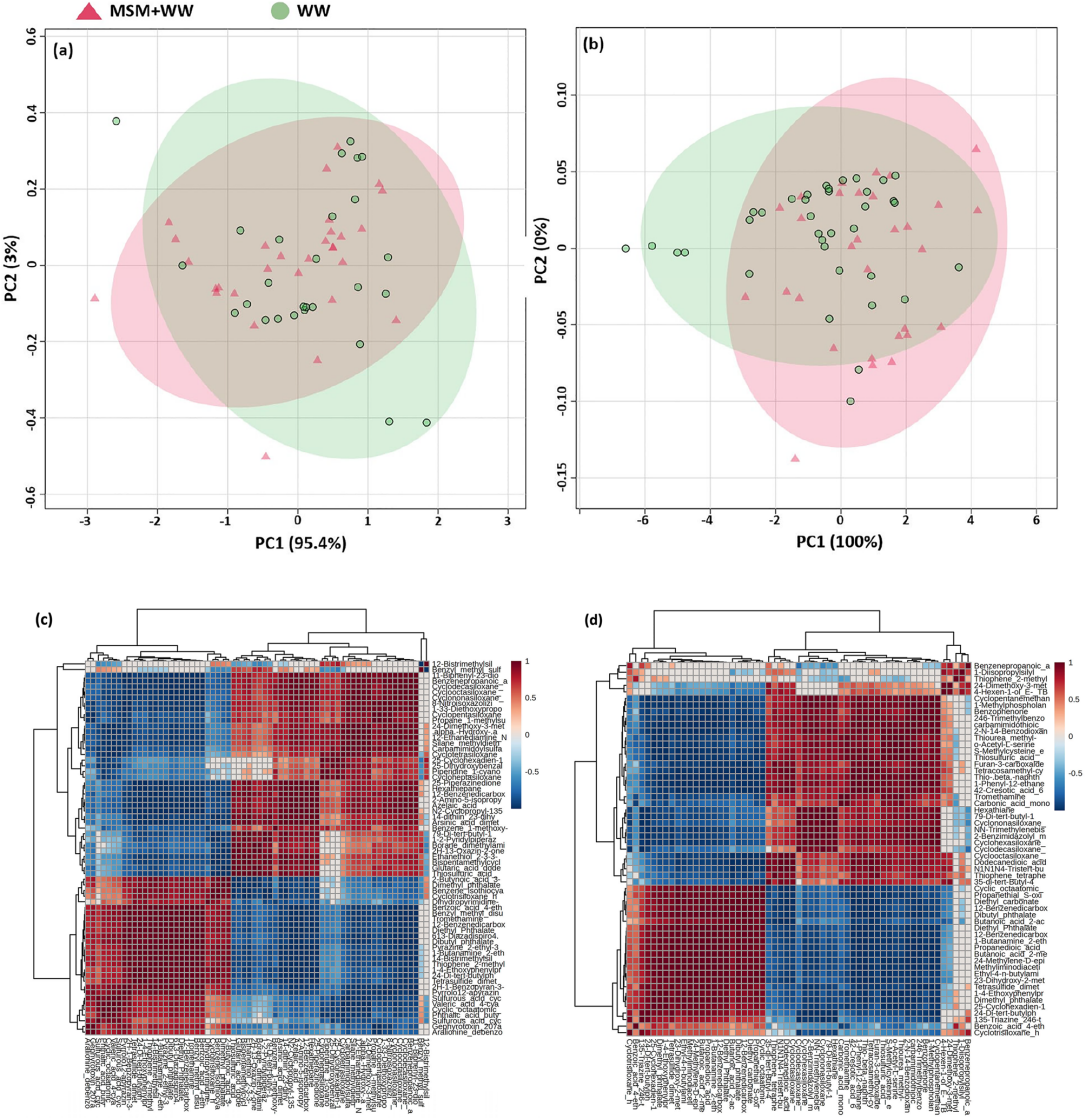
Score plot of (**a**) EC and (**b**) IC product quantification and correlation matrix of (**c**) EC and (**d**) IC fraction of MSM + WW and WW media.

FT-IR detected the functional groups, and GC–MS determined the resultant metabolites with a match quality of up to 99% of the mass spectral library. The GC–MS study revealed how the composition of the products formed altered after being exposed to flue gas. Using profiling, 37 and 29 metabolites were identified in the EC fractions, and 29 and 28 metabolites in the IC fractions for MSM+WW and WW media, respectively. In both media, the EC and IC fractions shared most of these metabolites, with few metabolites being specific to the MSM+WW and WW media. The metabolites were classified as carboxylic acids, carboxylic esters, fatty alcohols, and other sugars and amino acids. The GC–MS results were imported into the Metaboanalyst (v3.0) software for analysis. For normalization, metabolites were log_10_ transformed, and data were scaled to mean-centered solely for scaling. To illustrate product formation and reduce the dimensionality of the data acquired from flue gas bio-mitigation experiments, a multivariate analysis employing principal component analysis (PCA) was conducted. Using the 2D score plot and correlation matrix, the variation in EC and IC fractions between MSM+WW and WW media was depicted in Fig. 13. The PCA 2D score plot for the EC (Fig. 13a) and IC (Fig. 13b) fractions revealed statistically significant data clustering. Clustering and significant disparities between the groups ensured metabolic profile discrepancies. The semi-transparent oval in the center of the score plot reflects confidence intervals of 100 and 95.4% for the EC and IC fractions of the samples, respectively. The plot overlap indicates that the bacteria grown in both media create the same group of metabolites. It was determined that identical compounds were formed in both media, with significant changes in concentration. The bacteria rearranged its physiological functioning and synthesized many value-added compounds to survive under the stress of flue gas (CO_2_, NO, and SO_2_).

The metabolites identified in the EC fraction constituted unsaturated monocarboxylic acid, aromatic carboxylic acid, and fatty acids, such as 2-butynoic, benzoic, azelaic, valeric, phthalic, glutaric, 1–2 benzene dicarboxylic, arsinic acid, etc. It also produced fatty acid esters, such as butyl methyl, 4-cyanophenyl, hexadecamethyl, pentadecyl, ocatdecyl, decamethyl, tetradecamethyl, eicosamethyl ester, etc. Similarly, in the IC fraction, the metabolites identified had aromatic carboxylic acid and fatty acids, such as dodecanedioic, benzene propanoic, butanoic, propanedioic, methyliminodiacetic, carbonic, benzoic, 1–2 benzene dicarboxylic, carbamimidothioic acid etc. It also produced fatty acid esters, such as butyl methyl, dimethyl phthalate, hexadecamethyl, pentadecyl, ocatdecyl, eicosamethyl ester, etc. The GC–MS revealed numerous fatty alcohols and esters obtained in this study for application in the medicinal, cosmetic, food, and biofuel industries^24^. In Fig. 13c and d, using Pearson’s correlation test, a correlation matrix was built to calculate the correlation coefficients between the metabolites. The red area suggests a positive association between the metabolites, whereas the blue area indicates a negative correlation. All the groups in the EC and IC fractions are positively correlated, demonstrating that MSM+WW and WW media promote the synthesis of similar metabolites. The data indicated that the bacteria responded similarly to flue gas stress regardless of the media. The product synthesis concentration was deficient in the WW media due to less biomass development. The details of the compounds mentioned in Fig. 13c and d are presented in Supplementary Tables S1 and S2.

### Transport phenomena characteristics of the bio-mitigation process

In bioprocesses, chemical species undergo both transport and biological reactions simultaneously. The transport of substrates to cells occurs faster than metabolic biochemical reactions^30^. In aerobic processes, the gaseous mixture of O_2_, NO, SO_2,_ and CO_2_ is transferred from gas bubbles into the liquid phase and inside the cell. The mass and heat transfer characteristics obtained for the present bio-mitigation experiment conducted for the MSM+WW and WW media are depicted in Table 6. As CO_2_, O_2_, and NO are sparsely soluble in water, the gas–liquid interface presents the largest resistance on the liquid side. Due to the excellent solubility of SO_2_ in water, only the gas phase resistance is considered. The higher the gas solubility, the higher its saturation concentration in the media. The absorption rate was utilized to determine the respiration and gas uptake rates of the growing bacteria in the reactor. The gas uptake rates of NO and SO_2_ were identical in both media; however, the CO_2_ uptake rate in WW media decreased significantly. It was evident that the carbon intake by the bacteria for their growth was almost negligible; as a result, they did not exhibit any significant growth in the WW media. The respiration rate was lower in the MSM+WW medium due to the increased intake of other growth-promoting gases.

**Table 6:** Mass and heat transfer characteristics of the gaseous mixture in the aqueous media.

Parameters/media	Gas	Specific gas uptake rate (q_o_)	Gas uptake rate by bacteria	Gas transfer rate from gas to liquid	Saturation concentration (C*)	Volumetric mass transfer coefficient ($k_{L} a$/$k_{G} a$)	Heat of reaction ($\Delta H_{rxn}$)
		h^−1^ × 10^–3^	g L^−1^ h^−1^ × 10^–3^	g L^−1^ h^−1^ × 10^–3^	g L^−1^	h^−1^	kJ h^−1^
Minimal Salt Media + Wastewater	CO_2_	0.01	0.9	1.3	0.018	0.082	0.71
	NO	0.12	1.1	6.7	0.427	0.058	8.66
	SO_2_	3.9	34.8	–	–	0.01	206.33
	O_2_	0.01	0.09	0.4	0.030	0.031	200.83
Wastewater	CO_2_	0.005	0.0005	0.3	0.007	0.085	0.004
	NO	1	1.1	1.2	0.553	0.065	8.66
	SO_2_	3.4	3.5	–	–	0.002	206.70
	O_2_	2.1	2.2	3.2	0.637	0.00005	202.40

The rate of gas solute transfer from the bubbles to the aqueous medium is always faster than the rate at which the bacteria absorb the solute. Hence, the gas transfer rate in both mediums is higher than the uptake rate. The volumetric mass transfer coefficient gives a quantitative estimate of the effects of dissolved gas in the medium on the aeration efficiency of a bioreactor^73^. The $k_{L} a$ value in the MSM+WW media varies at 0.082, 0.058, 0.01, and 0.031 h^−1^ for CO_2_, NO, SO_2_ and O_2_, respectively. Similarly, for WW media, it varies by 0.085, 0.065, 0.002, and 0.00005 h^−1^ for CO_2_, NO, SO_2,_ and O_2_ in a bioprocess depending upon the solution viscosity, bubble coalescence tendencies, gas solubility, and diffusivity^30^. Absorption of a gas-phase solute into a liquid and subsequent chemical reaction alters the concentration profiles of the absorbed species and may increase the absorption rate. The concentration profile of gas absorption is affected by the gas's solubility and metabolic activity. While the consumption of NO and SO_2_ by the cells results in the same rate of heat evolution in both media, the consumption and heat evolution of CO_2_ decreases in the WW medium. Cell culture media are dilute aqueous solutions with near-ideal behavior. Hence, changes in mixing temperatures have little effect on the medium's composition as substrates are consumed and products are created. Since there is only a minor change in sensible heat, any temperature changes between the input and exit streams are unlikely to have much of an impact on the enthalpy. Therefore, the energy effect of reaction heat is considered^73^.

### Techno-economic assessment for flue gas bio-mitigation process

In addition to operating and equipment expenditures, a commercial facility incurs various other costs. Multiple variables can be used to establish a direct association between most of these expenditures. Based on the simplified cost model, Table 7 summarizes the techno-economic analysis of the total flue gas bio-mitigation for the MSM+WW and WW media^74^. The working capital of the process was computed on a reactor run basis of 12 days for a 20 L reactor for MSM+WW and WW media. Working capital measures the resources needed to carry out the process. A breakdown of the overall working capital includes costs of raw materials such as the MSM (commercial grade chemicals procured from IndiaMart, India), energy usage (US $ 0.096/unit for commercial use at BITS Pilani), and personnel compensation (salaries/labor charges at US $ 1.98/day at BITS Pilani). Based on the experimental run, the total working capital was projected to be $43.34 per reactor run for MSM+WW media and $42.37 for WW media for the 20 L reactor. For every reactor run with MSM+WW and WW media, the start-up expenses assigned to equipment modification as part of the capital investment were calculated to be $22.17 and $21.62, respectively.

**Table 7:** Analysis of TCI, FCI, and working capital for the 20 L bioreactor bio-mitigation process.

**Expenditure**		Cost of per reactor run (MSM + WW media)	Cost of per reactor run (WW media)
Working capital			
Raw materials	US$	0.97	0
Energy consumption	US$	18.50	18.50
Workforce	US$	23.87	23.87
**Total**	US$	43.34	42.37
**TCI (Total capital investment)**		**6.67 (Working capital)**	
	US$	289.08	282.61
**FCI (Fixed capital investment)**		**TCI−Working capital**	
	US$	245.74	240.24
**Direct cost**		**Onsite costs or Inside battery limits**	
Purchased equipment (PE) (30% of FCI)	US$	73.72	72.07
Installation (40% of PE)	US$	29.49	28.83
Instrumentation and control (18% of PE)	US$	13.27	12.97
Electrical equipment (14% of PE)	US$	10.32	10.09
		**Offsite costs or Outside battery limits**	
	US$	0	0
**Indirect costs**			
Engineering and supervision (12.5% of FCI)	US$	30.72	30.03
Contingency (12.5% of FCI)	US$	30.72	30.03
**Start-up costs**		**9% of FCI**	
	US$	22.17	21.62
Depreciation (DEP) (10% of FCI)	US$	24.57	24.02
Interest (INT) (3.5% of TCI)	US$	10.12	9.89
Tax + Insurance (0.03 FCI)	US$	7.37	7.21
Fixed charges (FC)		INT + DEP + Tax + Insurance	
	US$	42.06	41.12
Total product cost (TPC) (6.67 FC)	US$	280.54	274.27
Plant overhead (PO) (10% of TPC)	US$	28.05	27.43
Direct production cost (DPC) (60% of TPC)	US$	168.32	164.56
Manufacturing cost (MC)		DPC + FC + PO	
	US$	238.43	233.11
Revenue ((TPC−MC)/0.025)	US$	1684.4	1646.4
Profit before tax (Revenue−TPC)	US$	1403.86	1372.13
Profit after tax (0.52(Profit before tax−DEP))	US$	717.23	570.89
Cash flow (Profit after tax + DEP)	US$	741.8	594.91
Internal rate of return (IRR)$$\left( {\left( {\sqrt[{0.5}]{{\frac{cash flow}{{TCI}}}}} \right) - {1}} \right)$$	%	5.58	3.34
Net present value (NPV)$$\left( {\left( {\frac{cash flow}{{\left( {1 + IRR} \right)^{0.03} }}} \right) - {\text{TCI}}} \right)$$	US$	411.96	286.32
Payout time $$\left( {\frac{FCI + start - up}{{Profit after tax + DEP}}} \right)$$	Days	131	174
Benefit to cost ratio $$\left( {\frac{NPV}{{TCI}}} \right)$$		1.43	1.01

The investment in fixed capital equals the sum of direct and indirect costs. Expenses incurred at a specific location, as opposed to elsewhere, are referred to as ‘direct expenses’ and include the cost of supplies and labor utilized to install the appropriate machinery and wiring (inside battery limits). ‘Offsite costs’ refers to the expenditures incurred for constructing facilities required for a process located in a distant location (outside battery limits). The calculated onsite costs for this procedure are $126.80 for MSM+WW and $123.96 for the WW media reactor run. Engineering, monitoring, and budgeting are all examples of indirect costs that are often overlooked throughout the planning process. The calculated indirect costs for the procedure are $61.44 for MSM+WW and $43.24 for the WW media reactor run. So, the estimated fixed capital investment for the process was $245.74 per reactor run for MSM+WW and $240.24 per reactor run for WW. Total predicted capital expenditure for the process, including working capital and fixed capital expenditure, was $289.08 for an MSM+WW reactor run and $282.61 for the WW reactor run. The entire cost of this approach is equivalent to the sum spent on the bio-mitigation of flue gases and the manufacture of by-products with added value. Since MSM is required for optimal bacterial growth, improved flue gas mitigation, and wastewater remediation, its addition to the media resulted in a slight increase in the total capital investment (TCI). Increases in biomass production, bio-mitigation rate, and product formation were achieved with only a 0.6% increase in TCI due to the addition of MSM in the medium. The reactor incurred a working capital of $43.34 for the operation of the reactor for 12 days. Hence, the cost of operating the reactor per day to treat 20 L of wastewater and 2880 L of flue gas would be $3.6. The cost of treating per liter of wastewater and flue gas would be around $0.18 per day. Moreover, the direct production cost was estimated to be $168.32, and the cash flow was calculated to be $741.80. Based on these economic values, the internal rate of return, net present value, payout time, and benefit-to-cost ratio were calculated to be 5.58%, $411.96, 131 days, and 1.43, respectively. Therefore, the TEA shows that the process may be feasible for scale-up to the industrial level for the efficient remediation of flue gas and wastewater.

### NMR metabolomics and pathway analysis

**Figure 14:**
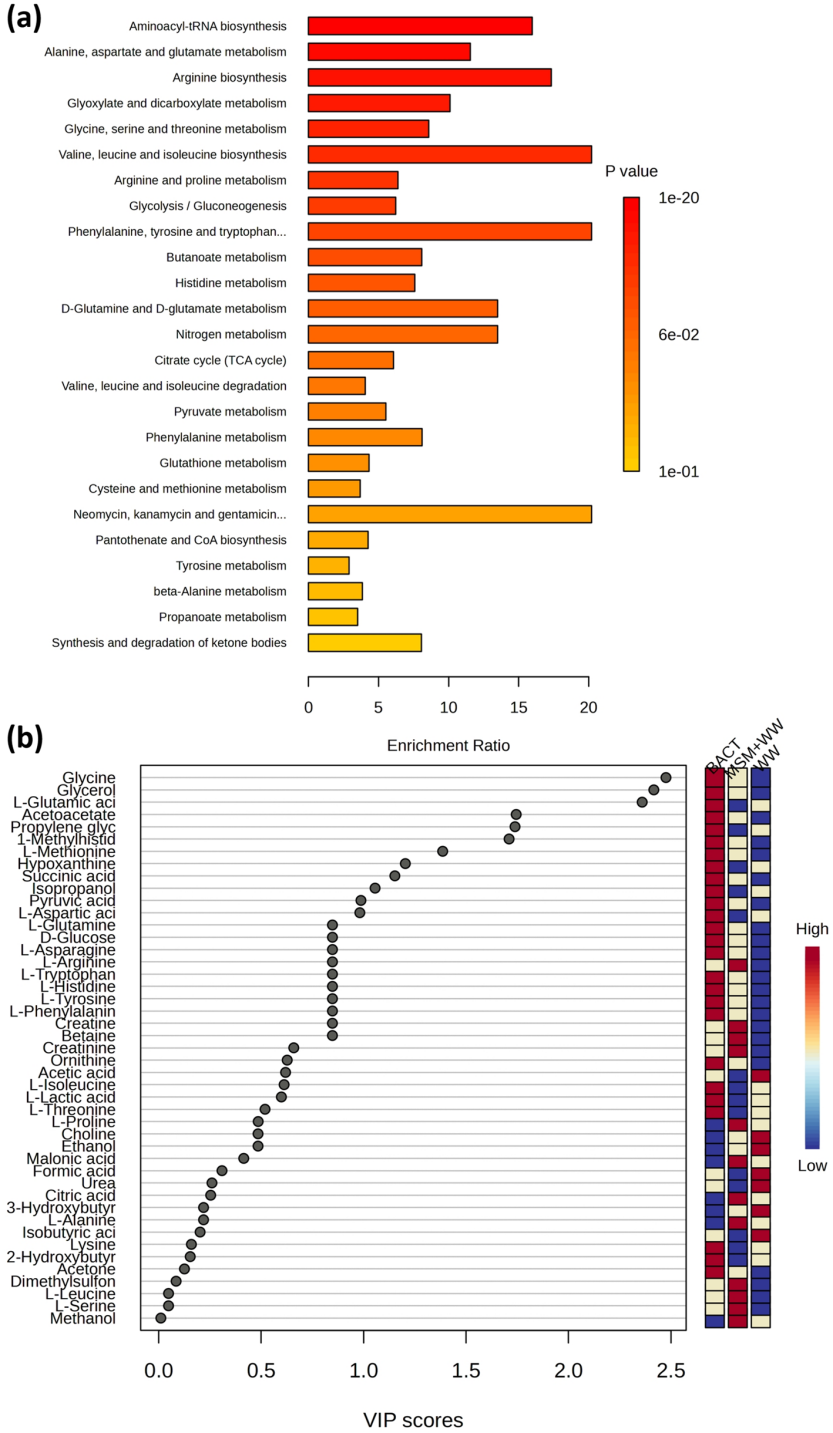
(**a**) VIP score plot of the bacterial cell constituents of inoculum, MSM+WW, and WW media and (**b**) enrichment analysis of bacterial pathways.

The NMR metabolomics approach was utilized to demonstrate the tolerance of bacterial cells to the stress of flue gas compounds and the cultivation media. The impact of the above factors on the bacterial cells was identified by various biophysical techniques such as VIP score plot (Fig. 14a), enrichment analysis (Fig. 14b), PCA biplot (Supplementary Fig. S3), heatmap correlation (Supplementary Fig. S4) and network correlation (Supplementary Fig. S5). The metabolomics approach defined how the flue gas stress influenced the regular bacterial metabolism and altered the physicochemical properties of the cells. The inoculum cells consisted of metabolites such as carnitine, creatine, choline, d-glucose, glycine, tyrosine, alanine, proline, threonine, asparagine, isoleucine, lysine, l-serine, l-arginine, creatinine, glutamine, leucine, valine, tryptophan, etc. These metabolites reflect the essential constituents of the bacterial cell. The VIP score plot reveals the alteration in the concentration of these metabolites in the cells under the stress of flue gas and cultivation medium (MSM+WW and WW). The red mark represents high, and the blue mark represents low concentration. The VIP score means the relative importance of the metabolite in the cells in the samples. A VIP score of >  = 1 is considered high importance, whereas a score of up to 1 is equally important. In the score plot, it is evident that in the inoculum cells, metabolites with a VIP score of >  = 1 are in high concentrations in the cells. Upon the introduction of the cells in the medium and reaction with flue gas, the constituents of the cells alter to a great extent, indicating a change in their composition. The concentration of the metabolites in the cells in the MSM+WW medium is high, whereas, in the WW medium, most of the metabolites fall too low concentration. This may be due to the biomass productivity of the bacteria, which is high in the MSM+WW media; hence, the bacteria alter accordingly to thrive in the media. However, in the WW, due to fewer nutrients available to the bacteria, it cannot thrive in the media, and very low alteration is depicted by the cells.

Similarly, by the concentration of the metabolites in the cell, the enrichment analysis describes the pathways followed to alter the metabolites in the cells. The enrichment ratio above 5 shows that most of the metabolite in the cells contributes to the particular metabolism pathway. In the result, all the metabolites identified positively correlate with the metabolism pathway, with a p-value ranging from 0.1 to 1^–20^, with 1^–20^ being the most relevant. The essential metabolism pathways of the bacteria in the bio-mitigation of the flue gas are RNA biosynthesis, alanine, valine, arginine, pyruvate, tyrosine, nitrogen, dicarboxylate metabolism, citrate cycle, gluconeogenesis, etc. The above results are also supported by the network correlation analysis, which represents the inter-dependency of the metabolites in the cells for the bio-mitigation flue gas. The correlation heat map defines the change in the concentration of the metabolites concerning the inoculum cells, which is higher (red) in the case of the MSM+WW media and lower (blue) for WW media. The PCA biplot is also represented to depict the alteration in the concentration of metabolites in the samples. The above biophysical techniques provide a clear understanding of the changes the bacterial cells undergo to cope with the environmental stress and, hence, mitigate flue gas. Therefore, it reflects the novelty of the work towards a better understanding of the biological functions of the bacteria characteristic to mitigation of CO_2_, NOx, and SOx.

### Thermodynamic assessment of bacterial growth and metabolism

Chemolithotrophic growth is a spontaneous and irreversible process. Bacteria obtain metabolic energy by facilitating the catalysis of inorganic chemical reactions that are not in a state of equilibrium in their surrounding environment due to kinetic factors, thereby promoting the progression of these reactions toward equilibrium^72^. The dissipation rate of chemical potential energy from the environment is contingent upon the efficiency of bacterial metabolism. The thermodynamic expenditure associated with biomass synthesis encompasses the comprehensive energy expenditure required for growth, converted into the energy content in biomolecules. Therefore, the cellular process can be conceptualized as a “black box” where inorganic compounds are introduced and subsequently transformed into biomass^75^. The method described involves integrating entropy production within an open system to facilitate the growth of a bacterial cell, as illustrated in Eq. (15). The temporal change in entropy within the cell can be expressed as the aggregate of all entropy fluxes exchanged with the surroundings and the rate at which entropy is generated through irreversible work (S_prod_). S_prod_ is positive, following the second law of thermodynamics, and serves as a genuine driving force for the process^76^.

15 $$\frac{dS}{{dT}} = \frac{{\dot{Q}}}{T} + \mathop \sum \limits_{i} \overline{{S_{i} }} n_{i} - \overline{{S_{x} }} n_{x} + \mathop {S_{prod} }\limits^{.}$$

$\frac{{\dot{Q}}}{T}$ denotes the entropy exchanged due to heat transfer to or from the cell with the environment, $\overline{{S_{i} }}$ is the partial molar entropy imported or exported through metabolites leaving or entering the cells, $n_{i}$ is the rate of molar exchange, where positive values indicate assimilation, $\overline{{S_{x} }}$ is the partial molar entropy of biomass formation, $n_{x}$ is the molar rate of newly formed biomass.

The minimum energy requirement for the biosynthesis of monomers such as alanine, asparagine, proline, glucose, etc., confirmed by NMR metabolomics, can be assessed by calculating Gibb’s energy for their formation from inorganic precursors (HCO_3_^−^, NH_4_^−^, H^+^, NO_3_^−^, SO_4_^2−^ etc.). The energy required to synthesize each monomer is calculated by Eq. (16)^77^.

16 $$\Delta G_{r} = \Delta G_{r}^{o} + RTlnQ$$

where, $\Delta G_{r}^{o} = \sum \Delta G_{products} - \sum \Delta G_{reactants}$, R is the ideal gas constant (0.008314 kJ K^−1^ mol^−1^), T is the absolute temperature (K), $Q = \frac{{\mathop \prod \nolimits_{1}^{n} product}}{{\mathop \prod \nolimits_{1}^{n} reactant}}$ is the reaction quotient (HCO_3_^−^ = 10^–2^, NO_3_^−^ = 10^–6^, SO_4_^2−^ = 10^–3^, H^+^  = 10^–7^, monomers = 10^–12^).

The standard Gibbs free energy value for synthesizing the monomers was determined by calculating the product's and reactant's energy change as documented in existing literature^69^. The present study involved the enrichment of bacteria in an aerobic setting, thereby establishing oxygen (O_2_) as the organism’s preferred electron acceptor (R_a_). The organism receives an electron donor (R_d_) in the form of S_2_O_3_^2−^ (Eq. 19), NO_2_^-^ (Eq. 21), or SO_3_^2−^ (Eq. 23) introduced via the MSM and gaseous mixture. The overall energy (∆Gr_0_) obtained from the electron transfer process between the donor and acceptor in the energy reactions (Re) is − 102.30, − 37.07, and − 129.02 kJ/e^−^ eq for S_2_O_3_^2−^, NO_2_^−^ and SO_3_^2−^, respectively. Therefore, Gibb’s overall free energy obtained for S_2_O_3_^2-^, NO_2_^-^, and SO_3_^2-^, as indicated in Eq. (22), is − 142.17, − 71.24, and − 146.11 kJ/e^−^ eq, correspondingly. The Gibbs free energy for energy reactions exhibits a negative value, indicating that the reaction is thermodynamically favorable at a specific temperature and is inclined towards product formation.

The energy reaction, R_e_, with S_2_O_3_^2−^ becomes: Re (Eq. 19) = Ra (Eq. 17)−Rd (Eq. 18)

17 $$\frac{1}{4} O_{2} + H^{ + } + e^{ - } \to \frac{1}{2} H_{2} O\;\;\Delta {\text{G}}^{0} = \, - {78}.{\text{72 kJ}}/{\text{e}}^{ - }$$

$$-$$

18 $$\frac{1}{8} S_{2} {O_{3}^{2 - }} + \frac{5}{8} H_{2} O \to \frac{1}{4} {SO_{4}^{2 - }} + \frac{5}{4}H^{ + } + e^{ - } \;\;\Delta {\text{G}}^{0} = { 23}.{\text{58 kJ}}/{\text{e}}^{ - }$$

19 $$\frac{1}{8} S_{2} {O_{3}^{2 - }} + \frac{1}{8} H_{2} O + \frac{1}{4} O_{2} \to \frac{1}{4} {SO_{4}^{2 - }} + \frac{1}{4}H^{ + } \;\;\Delta {\text{G}}_{{\text{r}}}^{0} = \, - {1}0{2}.{3}0{\text{ kJ}}/{\text{e}}^{ - }$$

The energy reaction, R_e_, with NO_3_^-^ becomes: Re (Eq. 21) = Ra (Eq. 17)−Rd (Eq. 20)

$$\frac{1}{4} O_{2} + H^{ + } + e^{ - } \to \frac{1}{2} H_{2} O\;\;\Delta {\text{G}}^{0} = \, - {78}.{\text{72 kJ}}/{\text{e}}^{ - }$$

$$-$$

20 $$\frac{1}{2} {NO_{2}^{ - }} + \frac{1}{2} H_{2} O \to \frac{1}{2} {NO_{3}^{ - }} + H^{ + } + e^{ - } \;\;\Delta {\text{G}}^{0} = { 23}.{\text{58 kJ}}/{\text{e}}^{ - }$$

21 $$\frac{1}{2} NO_{2}^{ - } + \frac{1}{4} O_{2} \to \frac{1}{2} {NO_{3}^{ - }} \;\;\Delta {\text{G}}_{{\text{r}}}^{0} = \, - {37}.0{\text{7 kJ}}/{\text{e}}^{ - }$$

The energy reaction, R_e_, with SO_3_^2-^ becomes: Re (Eq. 23) = Ra (Eq. 17)−Rd (Eq. 22)

$$\frac{1}{4} O_{2} + H^{ + } + e^{ - } \to \frac{1}{2} H_{2} O\;\;\Delta {\text{G}}^{0} = \, - {78}.{\text{72 kJ}}/{\text{e}}^{ - }$$

$$-$$

22 $$\frac{1}{2} SO_{3}^{2 - } + \frac{1}{2} H_{2} O \to \frac{1}{2} SO_{4}^{2 - } + H^{ + } + e^{ - } \;\;\Delta {\text{G}}^{0} = { 23}.{\text{58 kJ}}/{\text{e}}^{ - }$$

23 $$\frac{1}{2} SO_{3}^{2 - } + \frac{1}{2} O_{2} \to \frac{1}{2} SO_{4}^{2 - } \;\;\Delta {\text{G}}_{{\text{r}}}^{0} = \, - {129}.0{\text{2 kJ}}/{\text{e}}^{ - }$$

The biomass formula obtained from the CHNS analysis reported earlier for this study is C_6_H_13_O_15_N. Hence, the biomass synthesis reaction (R_e_) = the organic half-reaction for cell (Eq. 24)—Eq. (19)/Eq. (21)/Eq. (23). The findings suggest that the energetic aspects of biosynthesis are primarily influenced by the oxidation state of the environment rather than the chemical composition of the biomass. The energy requirements for biomass synthesis may differ for organisms growing under different conditions due to variations in biomass composition, temperature, and chemical environment. However, the impact of these factors is expected to be relatively minor compared to the influence of the oxidation state^76^. The energy invested in synthesizing biomolecules through thermodynamics constitutes a relatively minor proportion (10%) of the total energy expended during growth. The energy not directly converted into the Gibbs energy content of biomass during growth seems to be consistently related to the Gibbs energy needed for synthesizing the monomers^78^.

24 $$\frac{5}{4} CO_{2} + \frac{1}{4} NH_{4}^{ + } + \frac{1}{4} HCO_{3}^{ - } + H^{ + } + e^{ - } \to \frac{1}{4} C_{6} H_{13} O_{15} N + \frac{1}{2} H_{2} O$$

$$-$$

$$\frac{1}{8} S_{2} O_{3}^{2 - } + \frac{5}{8} H_{2} O \to \frac{1}{4} SO_{4}^{2 - } + \frac{5}{4}H^{ + } + e^{ - }$$

25 $$\frac{5}{4} CO_{2} + \frac{1}{4} NH_{4}^{ + } + \frac{1}{4} HCO_{3}^{ - } + \frac{1}{8} S_{2} O_{3}^{2 - } + \frac{3}{8} H_{2} O \to \frac{1}{4} C_{6} H_{13} O_{15} N + \frac{1}{4} SO_{4}^{2 - } + \frac{1}{4}H^{ + }$$

$$\frac{5}{4} CO_{2} + \frac{1}{4} NH_{4}^{ + } + \frac{1}{4} HCO_{3}^{ - } + H^{ + } + e^{ - } \to \frac{1}{4} C_{6} H_{13} O_{15} N + \frac{1}{2} H_{2} O$$

$$-$$

$$\frac{1}{2} NO_{2}^{ - } + \frac{1}{4} O_{2} \to \frac{1}{2} NO_{3}^{ - } + e^{ - }$$

26 $$\frac{5}{4} CO_{2} + \frac{1}{4} NH_{4}^{ + } \frac{1}{4} HCO_{3}^{ - } + \frac{1}{2} NO_{2}^{ - } + \frac{1}{4} O_{2} + H^{ + } \to \frac{1}{4} C_{6} H_{13} O_{15} N + \frac{1}{2} NO_{3}^{ - } + \frac{1}{2} H_{2} O$$

$$\frac{5}{4} CO_{2} + \frac{1}{4} NH_{4}^{ + } + \frac{1}{4} HCO_{3}^{ - } + H^{ + } + e^{ - } \to \frac{1}{4} C_{6} H_{13} O_{15} N + \frac{1}{2} H_{2} O$$

$$-$$

$$\frac{1}{2} SO_{3}^{2 - } + \frac{1}{2} O_{2} \to \frac{1}{2} SO_{4}^{2 - } + e^{ - }$$

27 $$\frac{5}{4} CO_{2} \frac{1}{4} NH_{4}^{ + } + \frac{1}{4} HCO_{3}^{ - } + \frac{1}{2} SO_{3}^{2 - } + \frac{1}{2} O_{2} + H^{ + } \to \frac{1}{4}C_{6} H_{13} O_{15} N + \frac{1}{2}SO_{4}^{2 - } \frac{1}{2} H_{2} O$$

## Conclusions

The research results of this study offer valuable knowledge on the simultaneous reduction of flue gas emissions and wastewater remediation using bacteria. Gaining insight into the function of environmental bacteria in this domain could be crucial for addressing ecological issues and advantageous for mitigating pollution. A direct relationship between the biomass production and removal efficiency of various inorganic compounds in the bioreactor system demonstrates the effectiveness of this process. Using a bubble column bioreactor in large-scale operations has demonstrated potential benefits, as indicated by its significantly higher biomass yield and improved removal efficiency for CO_2_, SO_2_, and NO. The primary mechanism responsible for the bio-mitigation of flue gas involves the synthesis of HCO_3_^−^, CO_3_^2−^, NO_3_^−^, SO_4_^2−^, and PO_4_^3−^ within the cultivation media. The study is the first of its kind to use NMR metabolomics investigation to facilitate the determination of the metabolic pathway employed by the bacteria to mitigate CO_2_, SO_2_, and NO and for biomass synthesis. The transport phenomena characteristics of the experimental system, as well as the thermodynamics assessment, indicated beneficial conditions for the metabolic biochemical reactions and subsequent product formation in the reactor. The techno-economic evaluation projected a capital investment as low as 20.5$ per day, which makes it a very efficient and economical technology. Thus, this study presents novel opportunities for the large-scale utilization of bubble column bioreactors and bacterial consortia in the bio-mitigation of flue gas, reducing environmental pollution while concurrently generating diverse value-added products.

### Supplementary Information

Supplementary Information.

## Data Availability

The datasets generated during the current study are available from the corresponding author upon reasonable request.
