# Supplementary material for: Sustainable synergistic approach to chemolithotrophs—supported bioremediation of wastewater and flue gas
Source: Sci Rep. 2024 Jul 17;14:16529. doi: 10.1038/s41598-024-67053-2 (PMC11254919; doi:10.1038/s41598-024-67053-2)
Supplement: Supplementary file 1 — Supplementary Information. [file 41598_2024_67053_MOESM1_ESM.pdf]

Sustainable Synergistic Approach to Chemolithotrophs - Supported Bioremediation of Wastewater and Flue Gas

Rachael J Barla[1], Suresh Gupta[1], Smita Raghuvanshi[1*]

[1]Department of Chemical Engineering, Birla Institute of Technology and Science (BITS), Pilani- 333031, Rajasthan, India

[*]Corresponding Author: Address: Faculty Division-1, Department of Chemical Engineering, BITS PILANI, Pilani – 333031, Rajasthan, India, Tel.: +91-1596-515638 (O), Email: smita@pilani.bits-pilani.ac.in

Supplementary

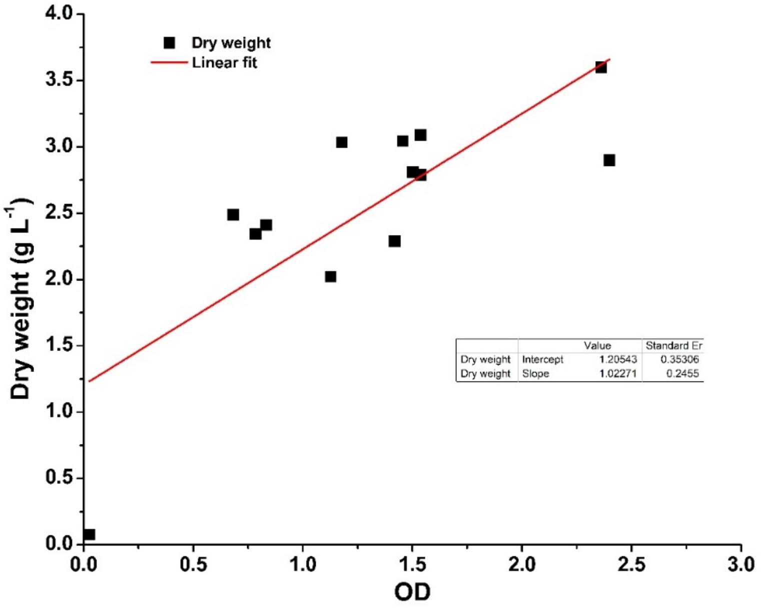

Fig. S1. The OD-dry weight curve obtained for the MSM+WW experiments.

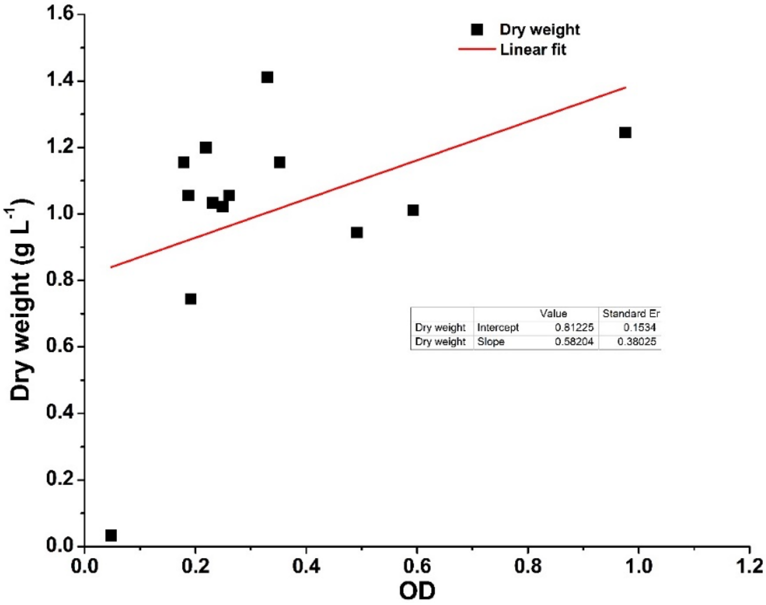

Fig. S2. The OD-dry weight curve obtained for the WW experiments.

Table. S1. The retention time details of the different metabolites identified in the EC fraction of MSM+WW and WW samples through GC-MS analysis.

Metabolite	Medium	Retention time (minutes)
Oxime-, methoxy-phenyl-_	WW	5.024
2-Butynoic acid, 3-trimethylsilyloxy-, methyl ester	WW	9.298
Tetrasulfide, dimethyl	WW	9.514
1-Butanamine, 2-ethyl-N-(2-ethylbutyl)-	WW	9.816
1,2-Ethanediamine, N,N'-dibutyl-	WW	10.044
8-Nitroisoxazolizidine	WW	10.37
1,2-Bis(trimethylsilyl)benzene	WW	11.125
2H-1,3-Oxazin-2-one, 3-[2-[dihydro-2-oxo-2H-1,3-oxazin-3(4H)-yl]ethyl]tetrahydro-	WW	11.315
Tromethamine	WW	15.022
Piperidine, 1-(cyanoacetyl)-	WW	15.92
1-(4-Ethoxyphenyl)propan-1-ol	WW	16.148
Pyrazine, 2-ethyl-3-(methylthio)-	WW	17.076
Benzoic acid, 4-ethoxy-, ethyl ester	WW	17.258
Aralionine, debenzoyl-	WW	18.408
2',4'-Dimethoxy-3'-methylpropiophenone	WW	18.512
Benzene, 1-methoxy-4-(methylthio)-	WW	18.595
1,4-Bis(trimethylsilyl)-1,3-butadiyne	WW	18.852
2,5-Cyclohexadien-1-one, 2,6-bis(1,1-dimethylethyl)-4-ethylidene-	WW	19.544
Gephyrotoxin 207a	WW	20.795
1,2-Benzenedicarboxylic acid, butyl methyl ester	WW	21.537
6,13-Diazadispiro[4.1.5.2]tetradecan-14-one	WW	21.768
N2-Cyclopropyl-1,3,5-triazine-2,4,6-triamine N6-acetyl	WW	22.011
Azelaic acid	WW	22.205
Borane, dimethylaminobis(2-methylaziridinyl)-	WW	22.468
Valeric acid, 4-cyanophenyl ester	WW	24.951
7,9-Di-tert-butyl-1-oxaspiro(4,5)deca-6,9-diene-2,8-dione	WW	25.752
2,5-Piperazinedione, 3,6-bis(2-methylpropyl)-	WW	26.047
Pyrrolo[1,2-a]pyrazine-1,4-dione, hexahydro-3-(2-methylpropyl)-	WW	26.277
Phthalic acid, butyl 2-methylpent-3-yl ester	WW	26.578
Glutaric acid, dodec-2-en-1-yl 3-nitrophenyl ester	WW	27.469
Cyclic octaatomic sulfur	WW	28.431
Cyclooctasiloxane, hexadecamethyl-	WW	28.921
2-Amino-5-isopropyl-8-methyl-1-azulenecarbonitrile	WW	30.973
2H-1-Benzopyran-3-carboxamide, N-[1-[4-(1H-imidazol-1-yl)phenyl]ethyl]-2-oxo-8-(2-propenyl)-	WW	31.239
[1,1'-Biphenyl]-2,3'-diol, 3,4',5,6'-tetrakis(1,1-dimethylethyl)-	WW	33.697
Sulfurous acid, cyclohexylmethyl pentadecyl ester	WW	48.004
Sulfurous acid, cyclohexylmethyl octadecyl ester	WW	53.374
Dihydropyrimidine-2-methyl thiosulfuric acid	MSM+WW	5.025
Carbamimidoylsulfanylacetic acid	MSM+WW	5.079
2,5-Dihydroxybenzaldehyde, 2TMS derivative	MSM+WW	6.405
Propane, 1-(methylsulfinyl)-	MSM+WW	6.587
Cyclotrisiloxane, hexamethyl-	MSM+WW	6.64
.alpha.-Hydroxy-.alpha.-methylbenzyl phenyl ketone	MSM+WW	6.812
1-(3,3-Diethoxypropoxy)-4-nitrobenzene	MSM+WW	7.158

Cyclopentasiloxane, decamethyl-	MSM+WW	7.358
Silane, methyldiethoxyisopropoxy-	MSM+WW	7.802
Benzene, isothiocyanato-	MSM+WW	9.1
Cyclotetrasiloxane, octamethyl-	MSM+WW	9.816
Benzyl methyl disulfide	MSM+WW	13.925
Dimethyl phthalate	MSM+WW	15.497
Arsinic acid, dimethyl-	MSM+WW	15.893
Cycloheptasiloxane, tetradecamethyl-	MSM+WW	16.033
2,4-Di-tert-butylphenol	MSM+WW	16.87
Thiophene, 2-[(methylthio)ethynyl]-	MSM+WW	17.071
Diethyl Phthalate	MSM+WW	18.777
Benzyl methyl sulfide	MSM+WW	19.965
Bis(pentamethylcyclotrisiloxy)hexamethyltrisiloxane	MSM+WW	21.333
Hexathiepane	MSM+WW	21.919
Ethanethiol, 2-(3-(3-chloro-2-pyridyloxy)propyl)amino-, hydrogen sulfate	MSM+WW	21.95
Thiosulfuric acid, S-[2-(2-hydroxy-1-indanylamino)ethyl]ester	MSM+WW	24.447
1,2-Benzenedicarboxylic acid, bis(2-methylpropyl) ester	MSM+WW	24.689
1-[2-Pyridyl]piperazine-4-thiocarboxylic acid 2-[1-2-pyridyl 1-oxide]	MSM+WW	25.502
Benzenepropanoic acid, 3,5-bis(1,1-dimethylethyl)-4-hydroxy-, methyl ester	MSM+WW	26.102
Dibutyl phthalate	MSM+WW	26.585
Cyclononasiloxane, octadecamethyl-	MSM+WW	31.404
Cyclodecasiloxane, eicosamethyl-	MSM+WW	35.854
1,4-dithiin, 2,3-dihydro-5-methyl-6-phenyl-	MSM+WW	47.069

Table. S2. The retention time details of the different metabolites identified in the IC fraction of MSM+WW and WW samples through GC-MS analysis.

**Metabolite**	**Medium**	**Retention time (minutes)**
Cyclotrisiloxane, hexamethyl-	WW	6.65
Diethyl carbonate	WW	8.299
1-Diisopropylsilyloxycyclohexane	WW	9.322
Tetrasulfide, dimethyl	WW	9.548
1,3,5-Triazine, 2,4,6-trimethoxy-	WW	12.702
Dimethyl phthalate	WW	15.493
1-(4-Ethoxyphenyl)propan-1-ol	WW	16.098
2,4-Di-tert-butylphenol	WW	16.867
Thiophene, 2-[(methylthio)ethynyl]-	WW	17.082
Cyclohexasiloxane, dodecamethyl-	WW	17.395
N1,N1,N4-Tris(tert-butyldimethylsilyl)succinamide	WW	17.667
Furan-3-carboxaldehyde, 2-methoxy-2,3-dihydro-	WW	18.151
Dodecanedioic acid, 2TBDMS derivative	WW	18.259
2',4'-Dimethoxy-3'-methylpropiophenone	WW	18.514
Diethyl Phthalate	WW	18.766
3,5-di-tert-Butyl-4-hydroxybenzaldehyde	WW	22.554
4,2-Cresotic acid, 6-methoxy-, bimol. ester, methyl ester, 4,6-dimethoxy-o-toluate	WW	23.281
2,4,6-Trimethylbenzoic acid, TMS derivative	WW	23.881

7,9-Di-tert-butyl-1-oxaspiro(4,5)deca-6,9-diene-2,8-dione	WW	25.75
2-Benzimidazolyl methane thiosulfuric acid	WW	25.811
N,N'-Trimethylenebis[s-3-aminopropylthiosulfuric acid]	WW	25.845
Benzenepropanoic acid, 3,5-bis(1,1-dimethylethyl)-4-hydroxy-, methyl ester	WW	26.099
Hexathiane	WW	26.212
Butanoic acid, 2-(acetylamino)-4-cyano-, 2,6-bis(1,1-dimethylethyl)-4-methoxyphenyl ester	WW	27.47
Cyclopentanemethanol, .alpha.-(1-methylethyl)-2-nitro-, [1.alpha.(S*),2.alpha.]-	WW	27.584
Thio-.beta.-naphthol cellobioside	WW	27.635
Cyclic octaatomic sulfur	WW	28.485
Cyclooctasiloxane, hexadecamethyl-	WW	28.924
Cyclotrisiloxane, hexamethyl-	MSM+WW	5.741
1-Butanamine, 2-ethyl-N-(2-ethylbutyl)-	MSM+WW	9.812
4-Hexen-1-ol, (E)-, TBDMS derivative	MSM+WW	10.71
Methyliminodiacetic acid	MSM+WW	10.865
Butanoic acid, 2-methyl-3-oxo-, ethyl ester	MSM+WW	10.94
Propanedioic acid, amino-, diethyl ester	MSM+WW	11.029
2,4-Methylene-D-epirhamnitol	MSM+WW	11.225
o-Acetyl-L-serine	MSM+WW	12.895
Thiosulfuric acid, 3-[2-[3-[1-phenyl-1H-tetrazol-5-yl]oxy]propyl]amino]ethyl ester	MSM+WW	12.832
Carbonic acid, monoamide, N-ethyl-, 2-ethylhexyl ester	MSM+WW	13.265
S-Methylcysteine, ethyl ester	MSM+WW	13.657
Propanethial, S-oxide	MSM+WW	14.38
2,3-Dihydroxy-2-methylpentanoic acid	MSM+WW	15.145
Thiourea, methyl-	MSM+WW	16.038
Tromethamine	MSM+WW	16.269
Benzoic acid, 4-ethoxy-, ethyl ester	MSM+WW	17.267
2,5-Cyclohexadien-1-one, 2,6-bis(1,1-dimethylethyl)-4-ethylidene-	MSM+WW	19.563
Benzophenone	MSM+WW	19.75
1,2-Benzenedicarboxylic acid, butyl methyl ester	MSM+WW	21.541
1-Phenyl-1,2-ethanediol, 2TBDMS derivative	MSM+WW	23.886
1,2-Benzenedicarboxylic acid, bis(2-methylpropyl) ester	MSM+WW	24.685
Ethyl-(4-n-butylamino)benzoate	MSM+WW	25.151
Cyclodecasiloxane, eicosamethyl-	MSM+WW	26.201
Dibutyl phthalate	MSM+WW	26.582
carbamimidothioic acid, N-[[(4-fluorophenyl)amino]thioxomethyl]-N'-2-propen-1-yl-, ethyl ester	MSM+WW	27.474
2-[N-[1,4-Benzodioxan-2-methyl]]aminoethanethiosulfuric acid	MSM+WW	28.557
1-Methylphospholane-1-oxide	MSM+WW	28.665
Cyclononasiloxane, octadecamethyl-	MSM+WW	31.408
Tetracosamethyl-cyclododecasiloxane	MSM+WW	38.197
Thiophene, tetraphenyl-	MSM+WW	46.911

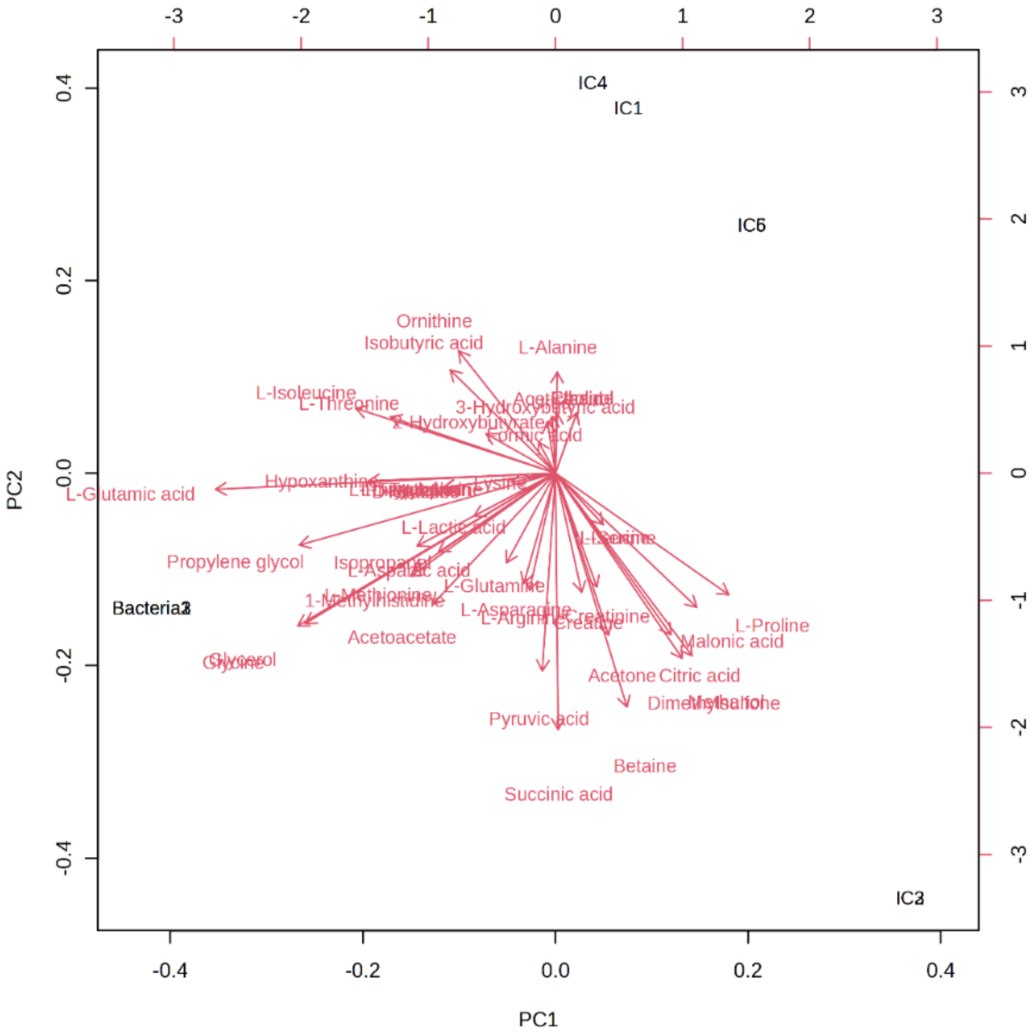

Fig. S3. The PCA bi-plot of the metabolites obtained in the IC fraction and inoculum of the MSM+WW and WW samples.

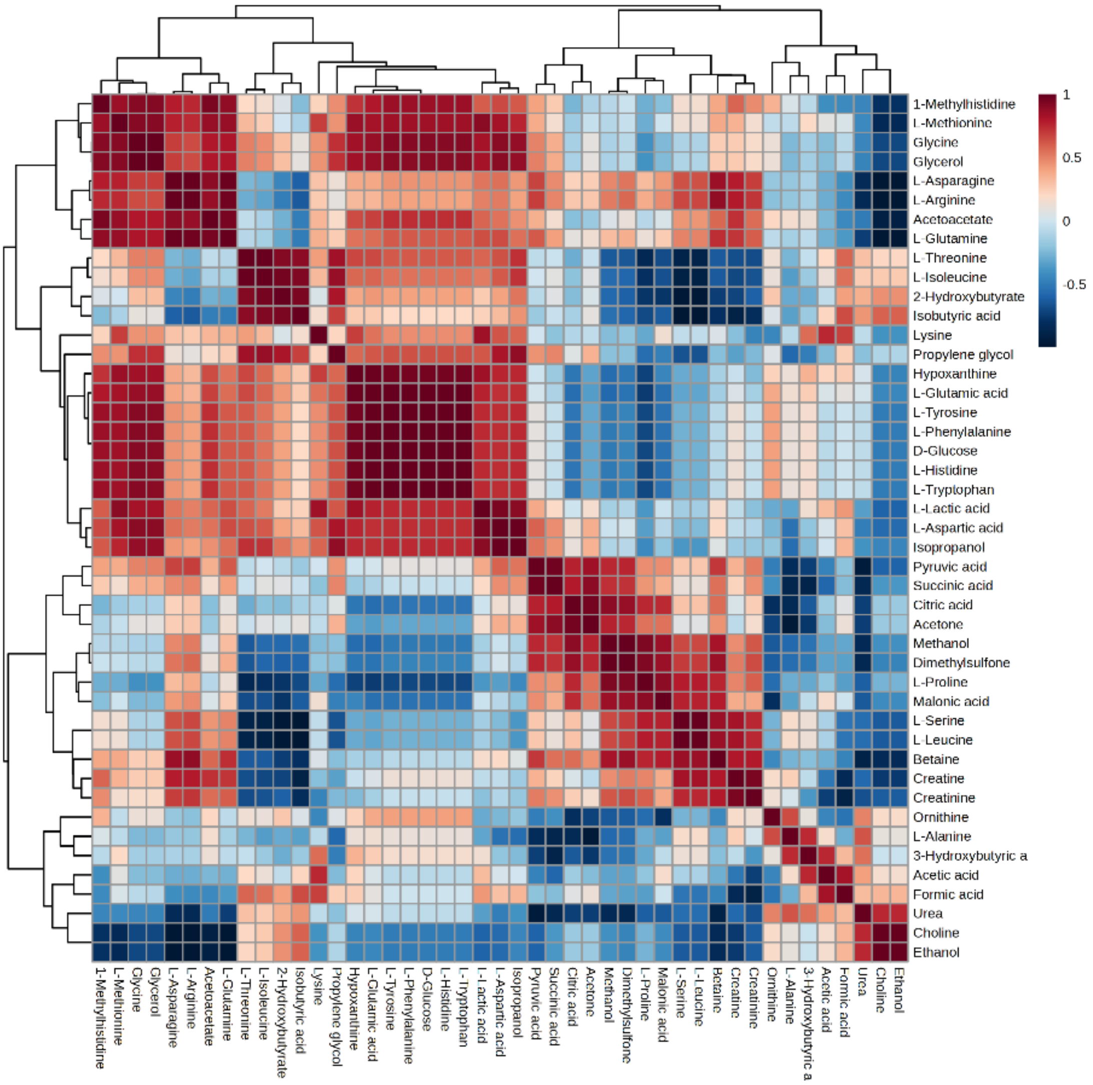

Fig. S4. The correlation heat map of the metabolites obtained for the MSM+WW and WW mediums.

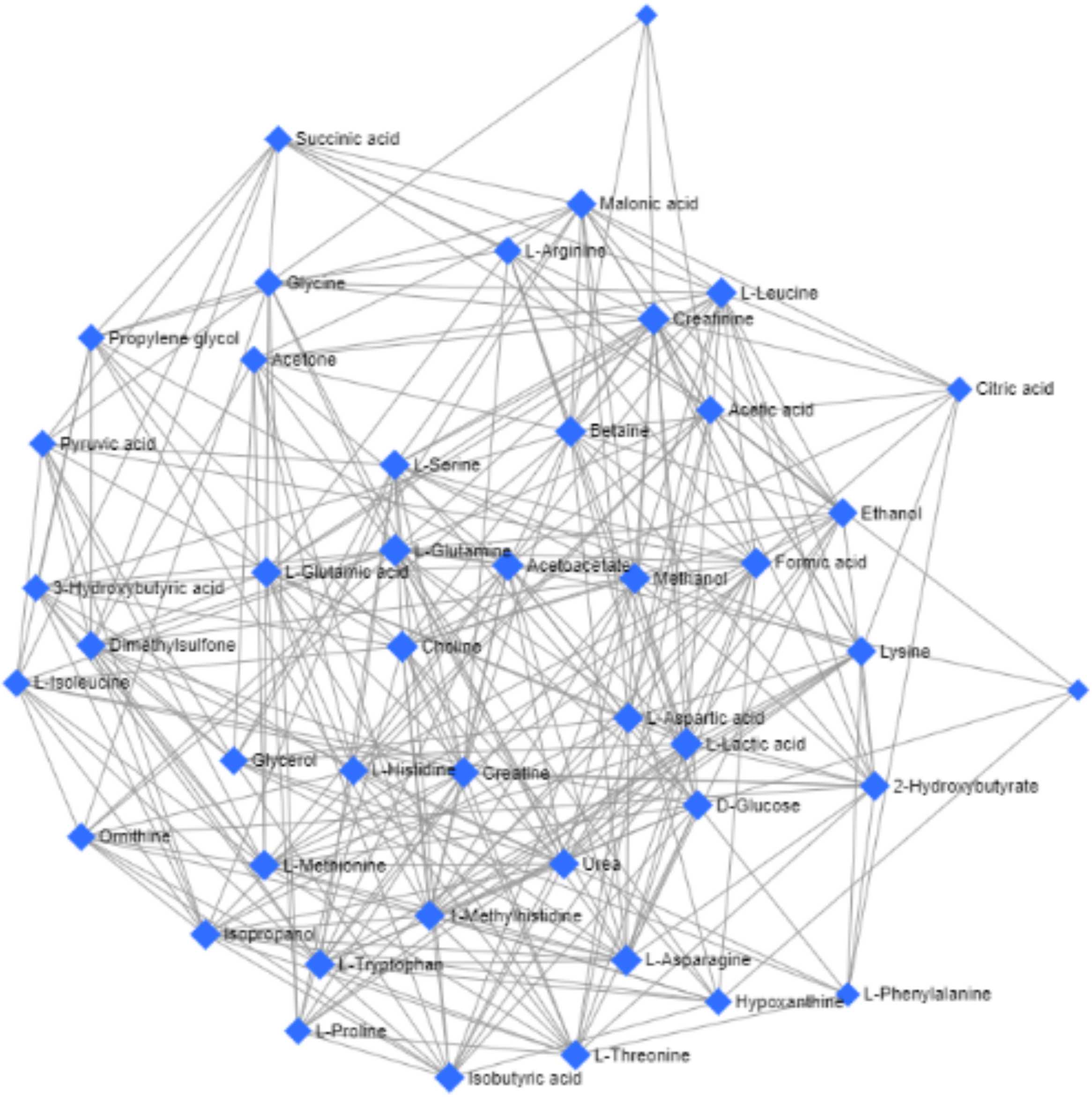

Fig. S5. The correlation network map between the metabolites for the MSM+WW and WW mediums following a metabolic pathway.
